# Formal Models of the Network Co-occurrence Underlying Mental Operations

**DOI:** 10.1371/journal.pcbi.1004994

**Published:** 2016-06-16

**Authors:** Danilo Bzdok, Gaël Varoquaux, Olivier Grisel, Michael Eickenberg, Cyril Poupon, Bertrand Thirion

**Affiliations:** 1 Department of Psychiatry, Psychotherapy and Psychosomatics, RWTH Aachen University, Aachen, Germany; 2 JARA-BRAIN, Jülich-Aachen Research Alliance, Germany; 3 Parietal team, INRIA, Neurospin, bat 145, CEA Saclay, Gif-sur-Yvette, France; 4 Institute of Clinical Neuroscience and Medical Psychology, Heinrich Heine University, Düsseldorf, Germany; 5 NeuroSpin, CEA, bat 145, CEA Saclay, Gif-sur-Yvette, France; University of Pennsylvania, UNITED STATES

## Abstract

Systems neuroscience has identified a set of canonical large-scale networks in humans. These have predominantly been characterized by resting-state analyses of the task-unconstrained, mind-wandering brain. Their explicit relationship to defined task performance is largely unknown and remains challenging. The present work contributes a multivariate statistical learning approach that can extract the major brain networks and quantify their configuration during various psychological tasks. The method is validated in two extensive datasets (n = 500 and n = 81) by model-based generation of synthetic activity maps from recombination of shared network topographies. To study a use case, we formally revisited the poorly understood difference between neural activity underlying idling versus goal-directed behavior. We demonstrate that task-specific neural activity patterns can be explained by plausible combinations of resting-state networks. The possibility of decomposing a mental task into the relative contributions of major brain networks, the "network co-occurrence architecture" of a given task, opens an alternative access to the neural substrates of human cognition.

## Author Summary

Assuming the central importance of canonical brain networks for realizing human cognitive processes, the present work demonstrates the quantifiability of relative neural networks involvements during psychological tasks. This is achieved by a machine-learning approach that combines exploratory network discovery and inferential task prediction. We show that activity levels of network sets can be automatically derived from task batteries of two large reference datasets. The evidence supports the often-held suspicion that task-specific neural activity might be due in large part to distinct recombinations of the same underlying brain network units. The results further discourage the frequently embraced dichotomy between exteroceptive task-associated versus interoceptive task-unspecific brain systems. Standard fMRI brain scans can thus be used to reconstruct and quantitatively compare the entire set of major network engagements to test targeted hypotheses. In the future, such network co-occurrence signatures could perhaps be useful as biomarkers in psychiatric and neurological research.

## Introduction

There is uncertainty about pertinent concepts of functional brain architecture. Systems neuroscience has established the existence of a set of fluctuating yet robust brain networks in humans [1, 2]. It however remains elusive how these neurophysiological phenomena relate to the repertoire of mental operations of an individual. This calls for methodological approaches that go beyond computing linear correlations (e.g., [3, 4, 5]) or independent components (e.g., [6, 7, 8]) in the "resting" human brain without controlled task modulation.

The central hypothesis of the present work is that network patterns can effectively describe fMRI data in both a resting mind-wandering and goal-directed task context. We introduce a methodological approach that enables formal assessment of this task-rest correspondance. On the one hand, the mostly *descriptive* statistical analyses used by many previous neuroimaging studies are extended in the present work by introducing an *inferential* statistical approach for the network involvement during task and at rest. On the other hand, the proposed approach combines deriving and predicting mutually overlapping *network patterns* (i.e., "network co-occurrence modeling"), whereas existing neuroimaging methods frequently focus on non-overlapping *voxel and region patterns*.

Identical neural networks have repeatedly been observed across cognitive domains using diverging neuroscientific methods. These observations prompted widely-adopted notions, including for instance the “default-mode network” [9], “salience network” [10], and “dorsal attention network” [11]. Such "networks" (i.e., spatiotemporally coherent signal patterns) are likely manifestations of electrophysiological oscillation structure [12, 13]. Developmentally, large-scale networks emerge during late fetal growth [8], before cognitive capacities mature in childhood. In adults, nodes of a same cohesive network probably have more similar functional profiles than nodes from different networks [14]. Indeed, resting-state fluctuations between large-scale networks were observed to be less stable than coupling between regions of each network when assessed by intra-class correlation [15]. Between-network connections were also less stable than intra-network connections when assessed by Kendall’s coefficient of concordance [15]. During task-unrelated random thought, the dynamical engagement of major brain networks therefore appear to be more volatile across participants and brain scans than intra-network dynamics [15–17]. While it is currently unknown "how global network architectures self-organize or reconfigure for specific tasks" [18], this hints at the *constellation of relative network involvements* as an under-appreciated unit of functional brain organization.

In line with this contention, the onset of a given cognitive task might induce characteristic changes in functional coupling of large-scale networks. For instance, the salience network and dorsal attention network tend to display blood-oxygen-level-dependent (BOLD) signal increases due to experimental stimulation, while the default-mode network often decreases across a wide range of tasks [19]. Whether stimulus-evoked compositions of such networks explain the majority [6] or only a fraction [4] of overall task activity is currently unresolved. For instance, a working-memory task entailed increase in BOLD activity in dorsal attention network regions but decrease in default-mode regions [20]. Notably, the functional connectivity did not change significantly within either dorsal attention network or default-mode network during this neuroimaging task. During auditory event transitions in another experimental fMRI study, both dorsal attention network and salience network increased in activity, whereas the default-mode network decreased in activity [21]. These changes of network constellation are probably mechanistically relevant for unfolding behavior [7, 22–24]. This idea is supported by evidence that proportional default-mode network recruitment impairs task performance, which is believed to be subserved by other large-scale networks [25, 26]. The mediation between canonical networks was tentatively proposed to involve the right anterior insula [21] and right temporo-parietal junction [27]. Moreover, the relevance of network engagement architectures possibly extends to psychiatric and neurological disorders [28, 29]. For instance, reciprocal coupling between default-mode network decreases and task-recruited networks were found to be absent in autism [30], reduced in schizophrenia [31] and major depression [32], as well as frequency-altered in attention deficit hyperactivity disorder [33].

The neuroarchitectural difference underlying the task behaviors and the idling brain has been investigated by what one can summarize as “dichotomy” and “manifold” hypotheses. The dichotomic view advocates functional antagonism between so-called “task-positive” and “task-negative” neural networks (e.g., [35]). Task-positive networks are believed to instantiate exteroceptive, environment-oriented mind sets to maintain task-constrained stimulus evaluation and response. Task-negative networks are believed to instantiate interoceptive, environment-detached mind sets to maintain adaptive mental imagery. The dichotomic view receives support from the following observations: a) the default-mode network consistently decreased in neural activity during many (not all) neuroimaging tasks [19, 34], b) activity in task-positive brain regions was consistently anti-correlated with activity in task-negative regions [35], and c) the spectrum of activity patterns in subcortical, limbic, and primary sensorimotor areas was found to be richer during task than at rest [4]. Hence, the dichotomic view predicts that extracting network components from resting-state data will yield a dictionary of network definitions insufficient to delineate task-specific network compositions.

In contrast, the “manifold” view advocates a fluctuating equilibrium of functionally distinct large-scale networks that is perturbed at task onset (e.g., [66]). This favors an identical ensemble of neural networks underlying functional brain architecture in focused and resting brain states. This view, in turn, receives support from the following observations: a) seed-region-based resting-state correlations frequently recovered task-typical networks [36], b) separate decomposition of task and rest activity maps yielded a number of topographically similar networks [6], and c) only 11% of whole-brain connectivity patterns always shifted at onset of different tasks [3]. Hence, the manifold view predicts that network components extracted from either rest or task data will perform similarly in capturing task-specific neural activity.

While the present investigation underlines the *functional integration* account of human brain organization, it will also be explicitly contrasted with the *functional segregation* account more frequently embraced by previous fMRI studies. Functional integration emphasizes brain function as an emergent property of complex connections between distinct brain regions [37, 38]. Functional specialization, in turn, emphasizes that microscopically distinguishable brain regions are responsible for solving distinct classes of computational processes [39, 40]. For instance, single-cell recordings and microscopic examination revealed the anatomical segregation of the occipital visual cortex into specialized V1, V2, V3, V3A/B, and V4 areas [41, 42]. Tissue lesion of the mid-fusiform gyrus of the visual system, as another example, is known to impair identity recognition from others' faces [43]. In neuroimaging research, specialized brain regions are frequently revealed by clustering algorithms [44, 45], such as k-means or hierarchical (ward) clustering. Functional brain network, however, are often extracted via matrix factorization methods [46], such as independent component analysis (ICA) and principal component analysis (PCA). It is important to appreciate that functional segregation findings necessitate neuroscientific interpretation according to non-overlapping, discrete region compartments, whereas network integration findings are to be interpreted by embracing cross-regional integration by overlapping network compartments. These considerations motivate the central question of the present study: To what extent do task-evoked fMRI signals lend themselves more to encoding and reconstruction in a network space rather than in a region space?

The present study systematically evalutes the possibility of analyzing fMRI tasks as co-recruitment of the entire set of major networks using multivariate statistical learning. A multi-step framework capitalized on two independent, large datasets (n = 500 and n = 81) with a total of 36 typical neuroimaging tasks. Each of these two task batteries attempted to cover the diversity of human cognitive processes. In an unsupervised learning step (i.e., naive to task labels), we first derived a small number of representative brain networks from these extensive neuroimaging resources. This first step yielded explicit models of possible network patterns with minimal statistical assumptions and without recourse to cognitive theory. In a supervised learning step (i.e., based on task labels), the dimensionality-reduced neuroimaging data were then submitted to an 18-task classification problem. For each task, this second step determined a plausible combination of the modes of variation in fMRI signals from the brain. This quantitative association with traditional psychological concepts made the data-driven results human-interpretable. In a validation step, we recovered task activation patterns from the respective component loadings in the learned statistical models. This third step evaluated how well formal network models can generate realistic task activation patterns. To show the usefulness of network co-occurrence modeling, we quantitatively revisited to what extent network activity underlying idling brain states can explain the variation of neural activity patterns during engagement in task performance.

## Results

### Overview

**Fig 1:**
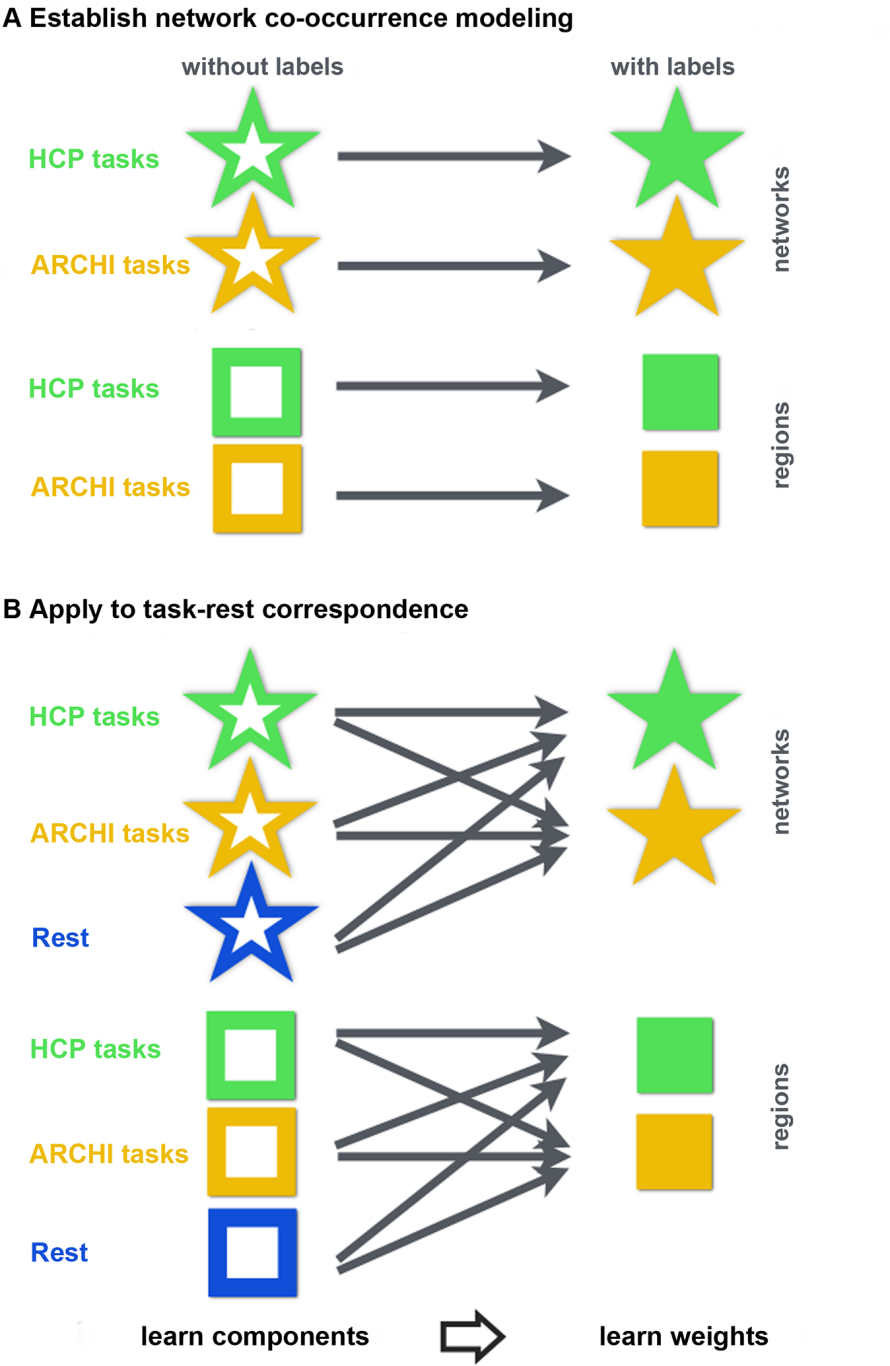
Overview of experimental analyses. The schematic summaries the modeling experiments undertaken in the present study. Symbols are introduced that describe how the HCP task data (*green*), the ARCHI task data (*yellow*), and the rest data (*blue*) were used to (*A*) evaluate the idea of network co-occurrence modeling and (*B*) test explicit hypotheses about the commonalities and differences between human brain activity in task-constrained and idling brain states [3, 6]. *Stars* indicate brain networks derived as mutually overlapping spatiotemporal patterns from independent component analysis (ICA), principle component analysis (PCA), sparse PCA, and factor analysis (FA). *Cubes* indicate discrete brain regions derived as mutually disjoint voxel groups from k-means and ward clustering. *Empty stars* or *cubes* indicate learning a network decomposition or region segregation from brain activity maps without the task labels (i.e., "unsupervised statistical learning"). *Filled stars* or *cubes* indicate learning a classification algorithm of 18 typical psychological tasks based on brain activity maps summarized as networks or regions with the task labels (i.e., "supervised statistical learning"). The origin of the *arrows* indicate from what data (HCP tasks, ARCHI tasks, or rest) the networks and regions were obtained. The *arrows* point to the set of psychological tasks that was captured by a predictive model based on the previously derived networks and regions. These symbols indicate the provenance of the results shown in the other figures.

**Fig 2:**
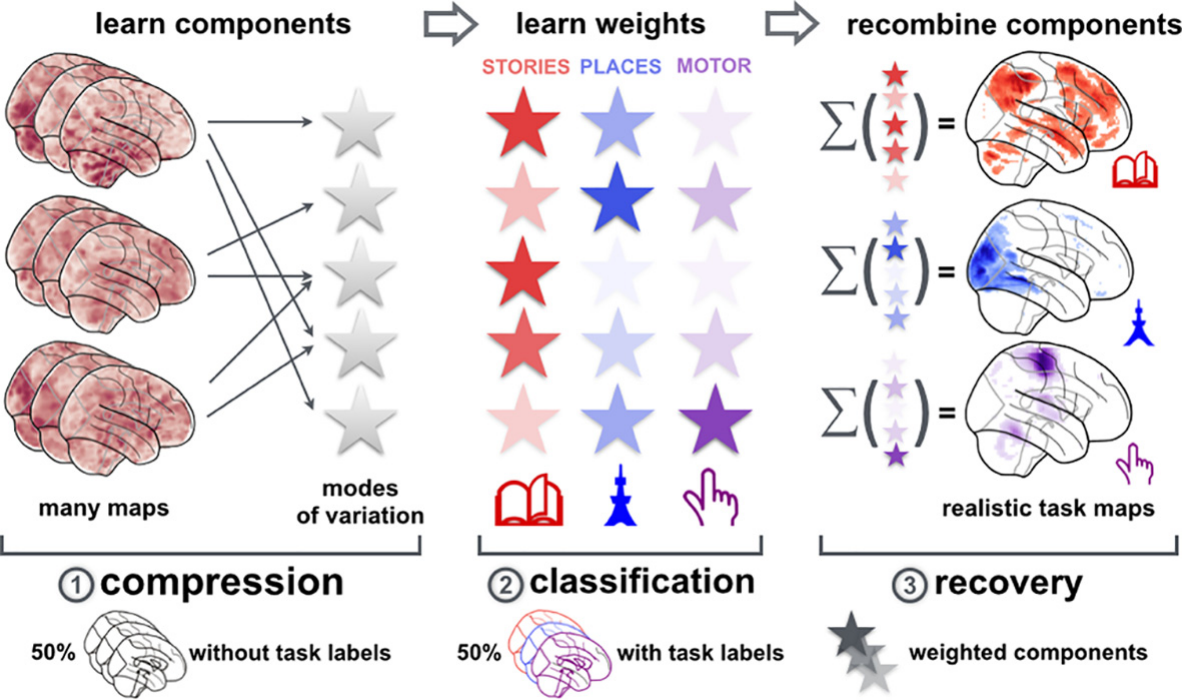
Network co-occurrence modeling: Workflow. The three steps of the proposed analysis approach are outlined. (*1*) In two large neuroimaging datasets (HCP with n = 500, ARCHI with n = 81), the spatial patterns of neural activity dominant across time series were discovered by data-driven decomposition of neural activity maps (first half of the data). The repertoire of major networks in the human brain was hence derived without access to what experimental task each activity map belongs. (*2*) This dictionary of explicit network definitions allowed reducing the remaining task activity maps (second half of the data) underlying traditional psychological concepts into 40 component loadings per neural activity map. Statistical learning based on these biologically motivated features found a linear model to distinguish 18 tasks by leave-one-participant-out cross-validation. A characteristic configuration of network engagements was thus automatically derived for each of 18 experimental tasks. (*3*) As face-validity criterion, task activity maps were generated from the weights of the trained classification models. These allowed quantifying the recovery performance of a given statistical model as a measure of biological meaningfulness of the learned model parameters (cf. methods section).

**Fig 3:**
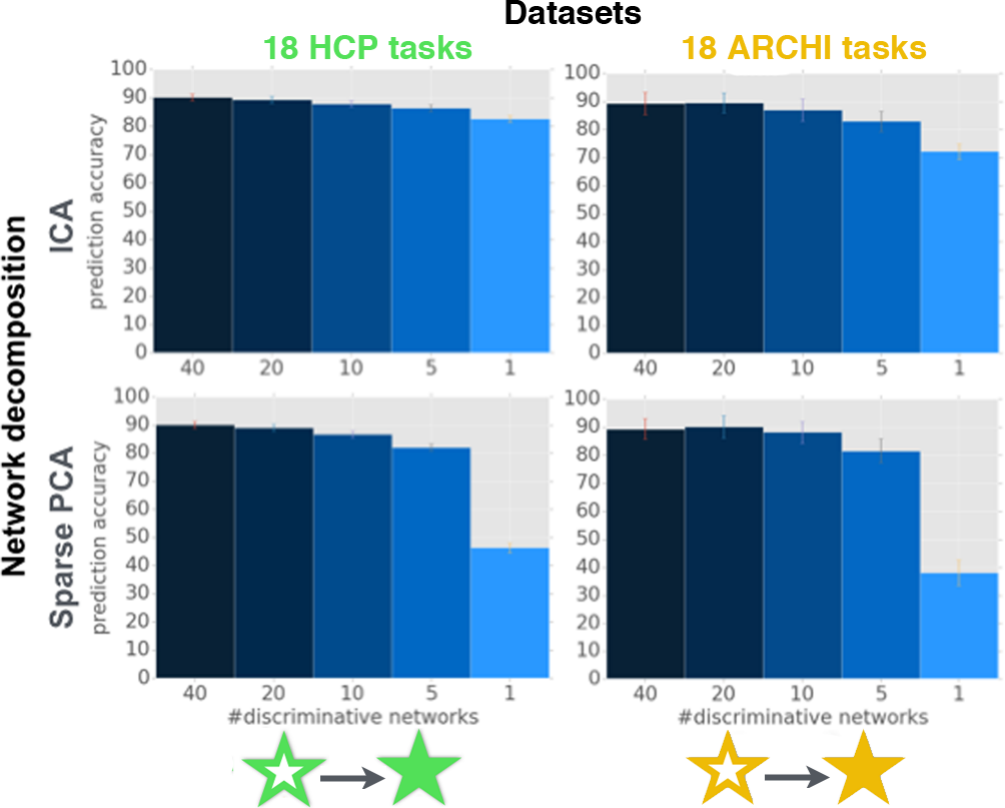
Network co-occurrence modeling: Predictive accuracy across network dictionary sizes. 40 ICA networks (*upper row*) and 40 sparse PCA networks (*lower row*) were discovered in HCP task data (*left column)* and ARCHI task data (*right column*) and used for feature engineering to facilitate classification of 18 psychological tasks (l2-penalized support vector machines, multi-class, one-versus-rest). One half of the task data (i.e., 4325 activity maps from HCP, 702 activity maps from ARCHI) were used for discovery of the ICA and sparse PCA networks. The network loadings of the previously unseen half of the task data (i.e., 4325 HCP maps, 702 ARCHI maps) were then submitted to an 18-task classification problem. The support vector machines were penalized by l2-regularization because classifier fitting was preceded by automatic selection of the k most relevant networks for each task (cf. methods section). We used a univariate feature selection procedure to evaluate the classification performance (*y axis*) as a function of k known network loadings per task (*x axis*). A two-step procedure therefore first subselected the k = 40, 20, 10, 5, and 1 most important network predictors for each task by univariate ANOVA tests and subsequent multivariate support vector machine fitting on the k most relevant network loadings per task. Note that each psychological task could therefore be associated with a different subselection of network loading features. To measure generalization performance, all task maps of one selected participant were left out in each cross-validation fold. See Fig 4 and S1–S3 Figs for the network topographies and the complete task-network assignments for each k.

**Fig 4:**
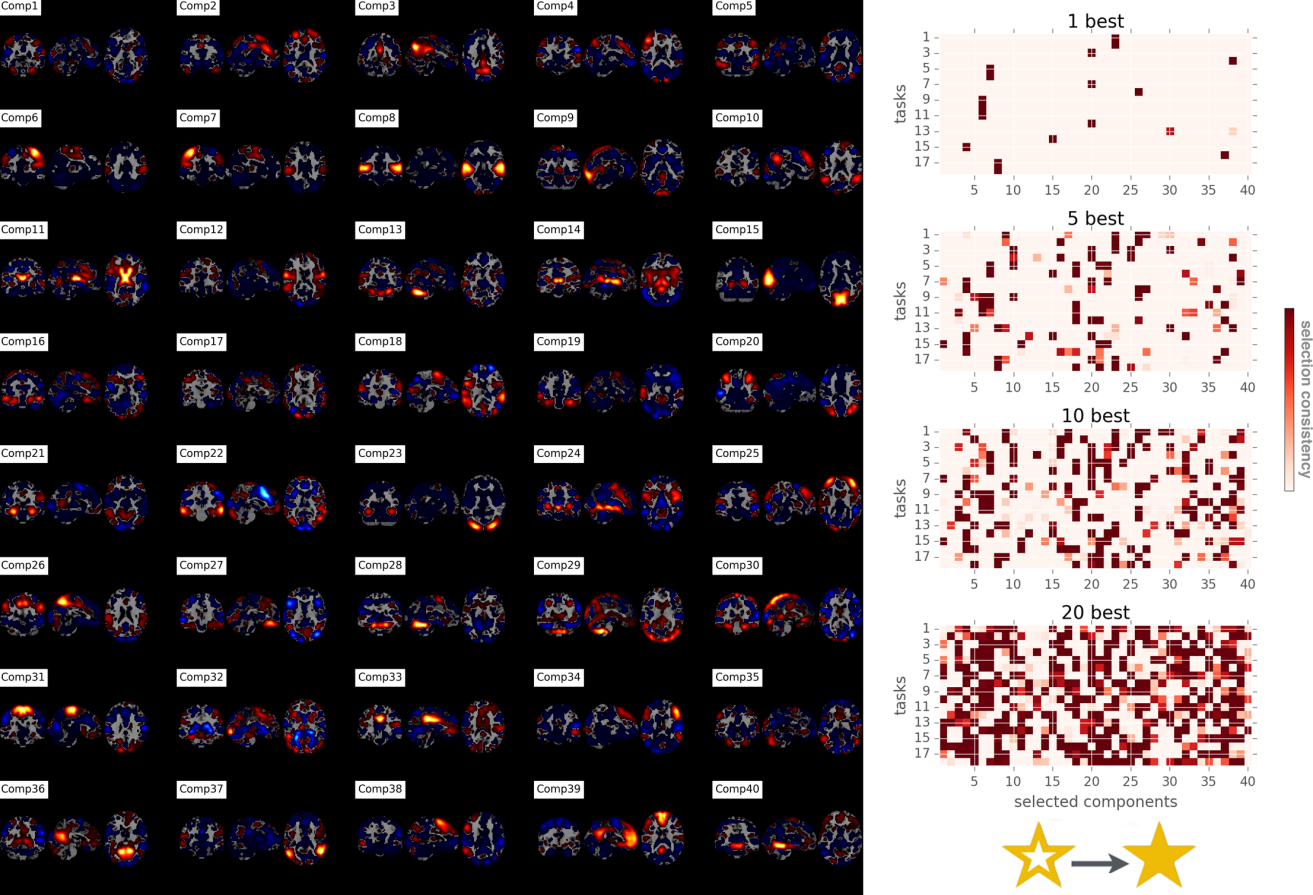
Network co-occurrence modeling: Sparse PCA network decomposition of ARCHI task maps and network-task assignment. 40 network components underlying 18 ARCHI tasks have been discovered by sparse PCA (*Comp1-40 on the left*). The ensuing network loadings from the second half of the ARCHI task data were submitted to classification of the psychological tasks based on the implication of brain networks (l2-penalized support vector machines, multi-class, one-versus-rest). l2-penalized support vector machines was employed to choose the most discriminative network variables by a preceding classical univariate test in a discrete fashion rather than by sparse variable selection based on l1 penalization (cf. methods section). This diagnostic analysis (*right*) revealed the most distinctive k = 1, 5, 10, and 20 network features (*red cubes*) for each experimental condition of the task battery (cf. Fig 3). The thus discretely selected network features per task were then fed into supervised multi-task classification as a feature space of activity-map-wise continuous activity values. The color intensity of the k cubes quantifies how often the corresponding brain network was selected as important for a task across cross-validation folds. This diagnostic test performed inference on a) the single most discriminative network for each task at k = 1, b) the network variables that are added step-by-step to the feature space of network implications with increasing k, and c) what network variables are unspecific (i.e., not selected) for a given task at k = 20. See tables for the corresponding descriptions of task 1–18. See S1–S3 Figs for analogous plots based on different matrix factorizations and datasets.

**Fig 5:**
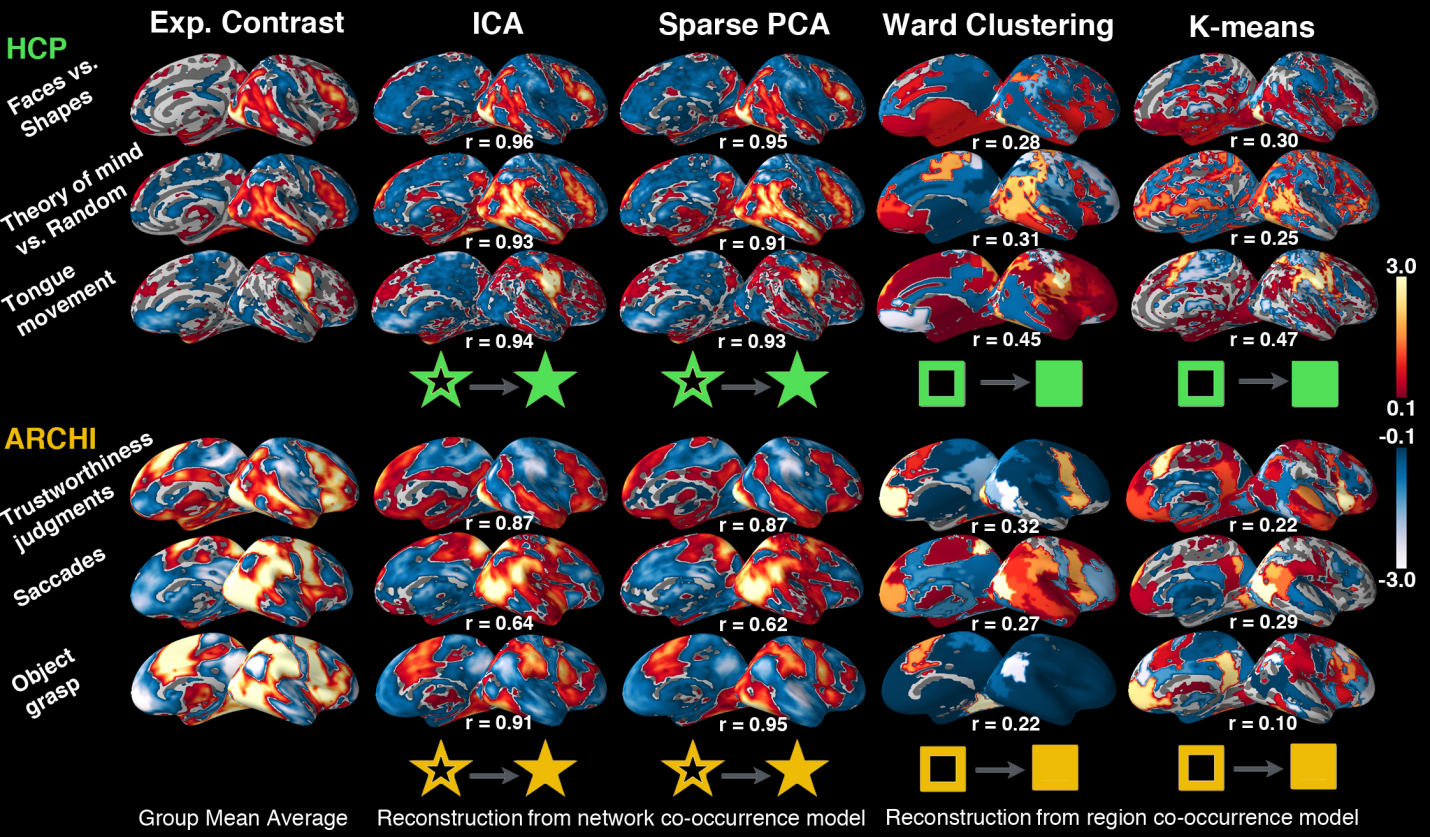
Network co-occurrence modeling: Comparing whole-brain reconstruction performance to region co-occurrence models. 40 networks from ICA or sparse PCA decomposition and 40 regions from ward or k-means clustering were discovered in HCP task data (*upper rows)* or ARCHI task data (*lower rows*) and used for classifying (l1-penalized support vector machines, multi-class, one-versus-rest) 18 psychological tasks in the remaining 50% of that same task data. For three exemplary tasks from HCP and ARCHI, the mean activity pattern across all participants is depicted (*leftmost column*). The corresponding whole-brain task activity derived from the network decomposition models ICA and sparse PCA capture proxies of functional brain networks by emphasis on functional integration (S4–S7 Figs). In contrast, task activity derived from the region parcellation models ward clustering (all region voxels are always spatially connected) and k-means clustering (no spatial constraint) capture proxies of functional brain regions by emphasis on regional specialization (S8–S10 Figs). The correlation values r quantify the voxel-wise similarity between the reconstructed activity map and the average activity map for each task and network decomposition method. This measure of recovery performance indicates the information loss incurred when first expressing activity maps as 40 network-wise loading values or 40 region-wise activity averages and then translating these values back into whole-brain space (cf. methods section). Consequently, learning network co-occurrence models outperformed region co-occurrence models in recovering realistic task activity, given an equal number of latent network and region components.

**Fig 6:**
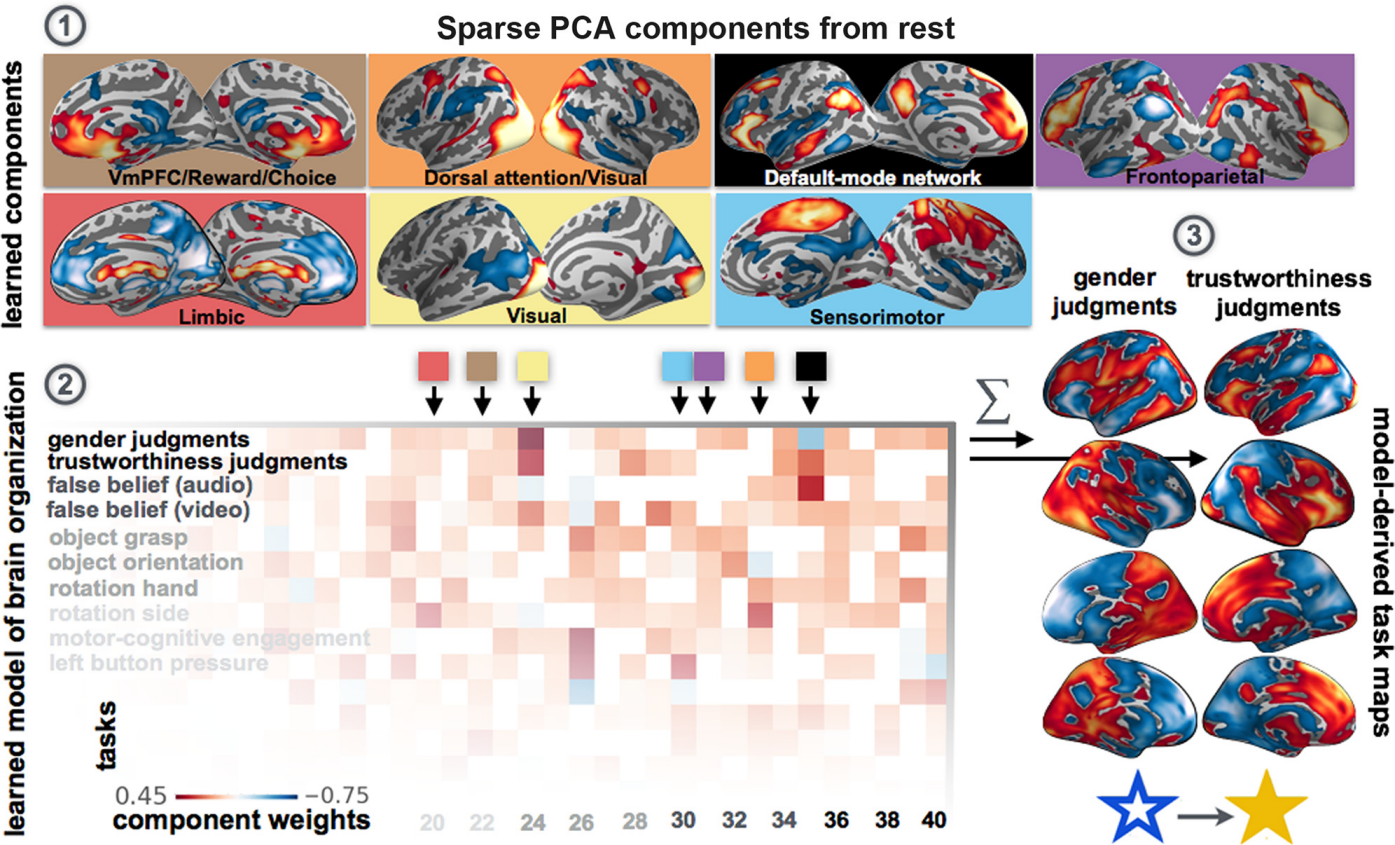
Task-rest correspondence: Composition of resting networks underlying psychological tasks. As a use case for network co-occurrence modeling, an insufficiently understood question of human brain organization has been quantitatively revisited: the correspondence between neural activity during goal-directed tasks and idling mind-wandering. 40 sparse PCA networks were revealed in rest data and used for supervised classification (l1-penalized support vector machines, multi-class, one-versus-rest) of 18 psychological tasks from the ARCHI task battery. (*1*) Seven examples from the 40 spatiotemporal activity patterns drawn from task-unrelated resting-state fluctuations using sparse PCA decomposition. This enabled translation of whole-brain task activity maps (>60,000 voxels) from the ARCHI task data into 40 network component loadings per activity map. (*2*) These measures of network implication served as basis for statistical learning of a sparse classification model that disambiguates activity maps from the 18 psychological task. In the depicted matrix, each square represents an automatically determined classification model weight that corresponds to the importance of one specific large-scale network (*x axis*) during a given cognitive task (*y axis*). l1-penalization of the classification algorithm induced zero model weights (*white*) for automatic variable selection of the resting-state networks that are specifically associated with a given task (*red or blue*), in contrast to discrete selection of the k best network features (Figs 3 and 4). (*3*) The network weights of the fitted model is exploited for de-novo generation of realistic whole-brain activity maps for each of the 18 tasks. This is exemplified by gender judgments and trustworthiness judgments on visually presented faces: Consistent with previously published experimental fMRI studies [53], the default-mode network (*black square*), implicated in higher-order social processing, exhibited significant increase with trustworthiness judgments but decrease with gender judgments on faces. Both face discrimination tasks rely on the visual cortex (*yellow square*), the limbic system (*red square*), and the reward- and choice-related ventromedial prefrontal cortex (*brown square*). The dorsal attention/visual network (*orange square*) was detected as non-discriminatory for the two facial judgments tasks (i.e., weight is zero). Further, the frontoparietal network with extensive dorsolateral prefrontal cortex implication (*purple square*) was only associated with gender judgments, whereas the sensorimotor network (*blue square*) was only associated with trustworthiness judgments.

**Fig 7:**
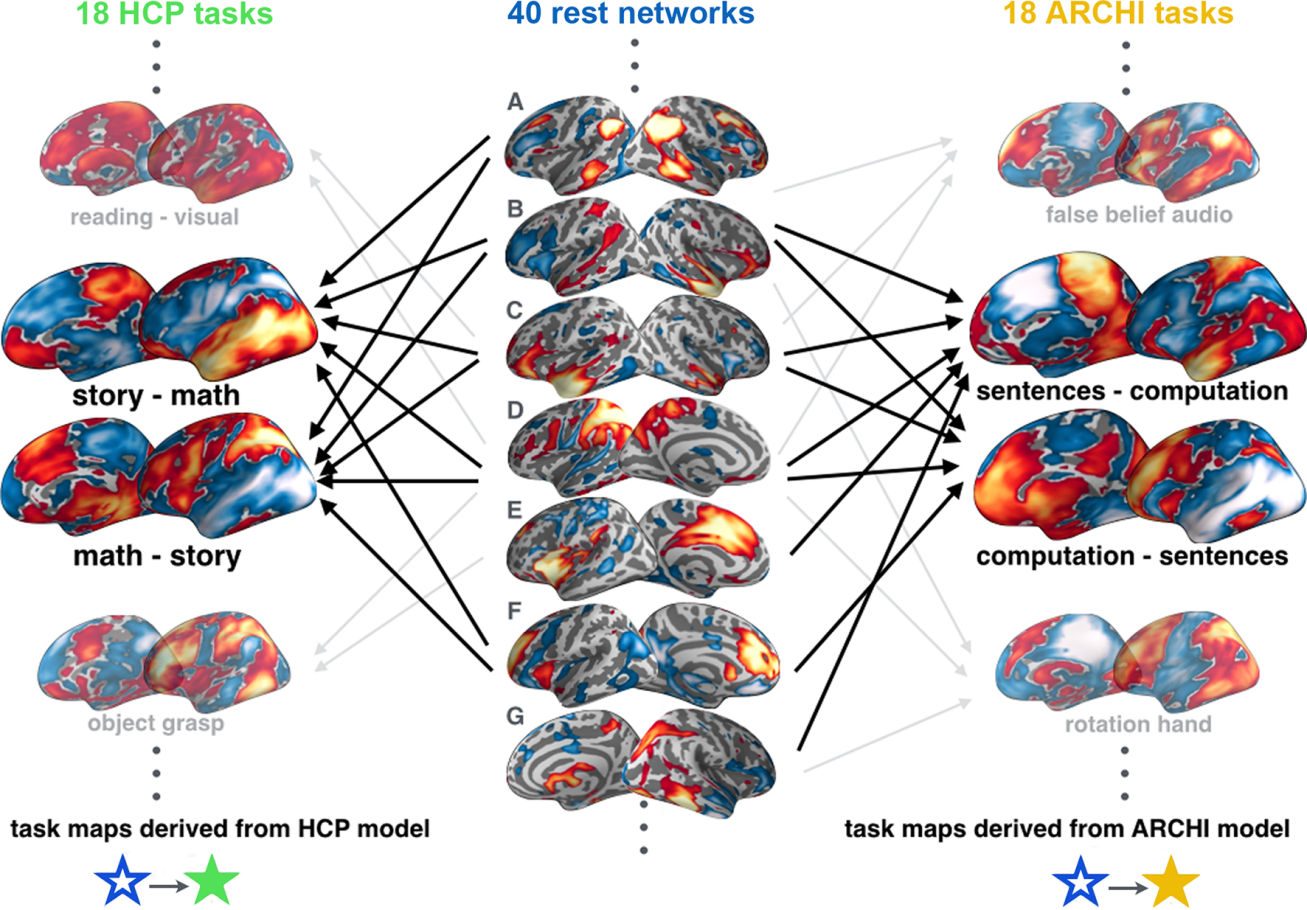
Task-rest correspondence: Reconstructing two similar tasks from two different datasets based on the same resting networks. 40 sparse PCA networks were discovered from the same rest data and used for feature engineering as a basis for classification (l1-penalized support vector machines, multi-class, one-versus-rest) of 18 psychological tasks from HCP (*left)* and from ARCHI (*right*). *Middle column*: Examples of resting-state networks derived from decomposing rest data using sparse PCA. Networks B and C might be related to semantics processing in the anterior temporal lobe [54], network D covers extended parts of the parietal cortex, while networks E and F appear to be variants of the so-called “salience” network [10]. *Left/Right column*: Examples of task-specific neural activity generated from network co-occurrence models of the HCP/ARCHI task batteries. *Arrows*: A diagnostic subanalysis indicated what rest networks were automatically ranked top-five in distinguishing a given task from the respective 17 other tasks (i.e., k = 5 analogous to analyses in Figs 3 and 4). Although the experimental tasks in the HCP and ARCHI repositories, “story versus math” and “sentences versus computation” were the most similar cognitive contrasts in both datasets. For these four experimental conditions the model-derived task maps are highly similar. Consequently, two independent classification problems in two independent datasets with a six-fold difference in sample size resulted in two independent explicit models that, nevertheless, generated comparable task-specific maps. This indicated that network co-occurrence modeling indeed captures genuine aspects of neurobiology rather than arbitrary discriminatory aspects of the data.

**Fig 8:**
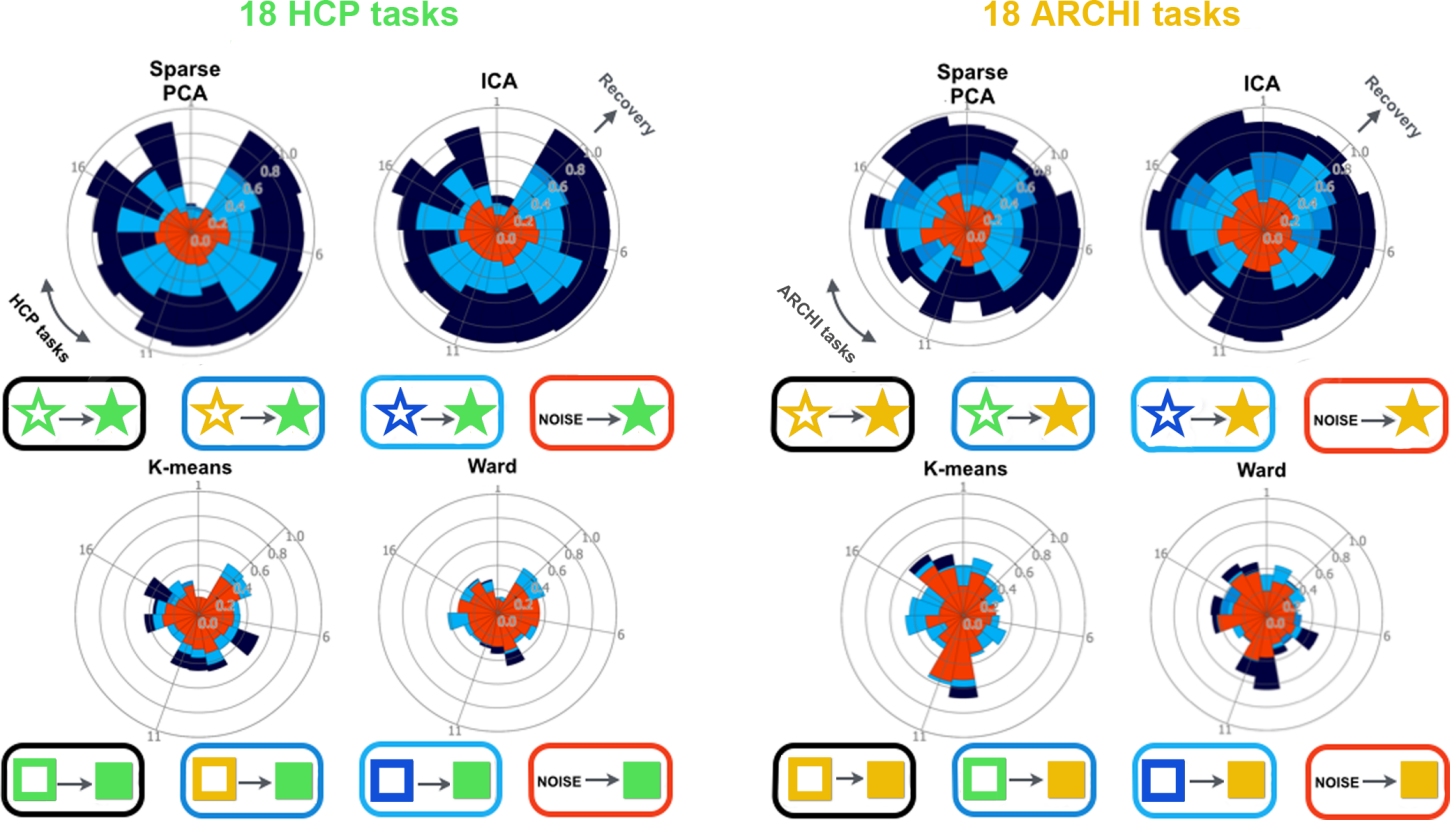
Task-rest correspondence: Recovery performance across network and region atlases. 40 networks (*upper row*) were discovered in independent component analysis (ICA) and sparse principal component analysis (sparse PCA). 40 regions (*lower row*) were derived from k-means and ward clustering based on four diverging types of neural activity data. Network and region atlases were derived from i) identical task-data as positive test (*dark blue*), ii) non-identical task-data (*medium blue*), iii) resting-state data (*light blue*), and iv) Gaussian noise as negative test (*red*). The ensuing networks and regions were then used to create a feature space of neural activity patterns for 18-task classification (l1-penalized support vector machines, multi-class, one-versus-rest) and subsequently measure the per-task recovery performance. The recovery performance of all 18 tasks (radial columns) is measured by the Pearson correlation between the model-derived task activity maps and the average first-level task map. As an important observation, network dictionaries derived from different tasks and from rest data were similarly successful in recovering whole-brain activity during diverging experimental tasks, while specialized regions achieved much worse recovery performances in both datasets. See S11 and S12 Figs for additional analyses.

The analyses, results, and figures are divided into two different sections (see schematic in Fig 1). In the first section (Figs 2–5, entitled "network co-occurrence modeling"), we statistically tested whether neural activity patterns measured with fMRI in humans can be largely explained by changes in cohesive network units. The term “network” henceforth refers to spatiotemporal modes of variation extracted from time series of fMRI activity whose weighted linear combination sum up to whole-brain activity patterns [6, 47, 48]. Whole-brain activity maps were expressed as a linear combination of 40 spatiotemporally coherent patterns (i.e., network components). The distributed BOLD signals from voxel space were thus reduced to 40 component loadings in a network space. The network engagement pattern is shown to successfully classify and restore neural activity from the 18 psychological tasks across two large datasets. In the second section (Figs 6–8, entitled "task-rest correspondence"), this possibility to compress neural activity maps into low-dimensional summaries of brain network involvements was used to revisit the relationship between the functional brain architecture during defined psychological tasks and unconstrained task-free mental activity. While the first section used a same task dataset (either HCP or ARCHI) for both unsupervised network discovery and supervised multi-task classification, the applied second section probed diverging ways of deriving the network dictionary for subsequent classification. In particular, networks from i) the same task data dataset, ii) the respective other task dataset, iii) rest data, and iv) Gaussian noise were quantitatively assessed for their commonalities and differences in restoring task activity patterns.

### Establishing an approach to quantifying the implication of network sets: Network co-occurrence modeling

We first assessed the predictive accuracy of network co-occurrence models across methodological choices and datasets (Figs 2–5). Without using any rest maps, ICA and sparse PCA were applied to 50% of the task maps to then translate the remaining 50% of task activity maps into sets of network loadings. l1-penalized support vector machines (SVM) with C-hyperparameter tuning on ICA and sparse PCA loadings (i.e., second 50% data) correctly detected 18 tasks 90% to 93% of the time in both HCP and ARCHI datasets (Fig 2). Note that l1 penalization in these and below classification model estimations increased interpretability by introducing zeros into the model weights corresponding to network loadings (cf. methods section). Note further that chance level is at 5.6% in an 18-class scenario. To evaluate diagnostic metrics typically used in machine learning [49] for ICA and sparse PCA, the *recall* (How many brain images labeled as a cognitive task were correctly recognized to belong to that class?) ranged between 90% and 93%, while the *precision* (How many brain images recognized to belong to a certain class were really labeled as that class?) ranged between 87% and 90%. We deemphasized results based on PCA and factor analysis whose lower accuracy scores ranged between 87% and 92%. Additionally, ICA and sparse PCA also yielded higher model sparsity (induced by l1-norm penalization to bias the SVM estimation) than PCA and factor analysis, computed by sparsity = ∑ [||weights||_1_ / ||weights||_F_] based on the vectorized SVM weights of all 18 classes. This parsimony-based metric of model "effectiveness" ranged between 8.55 and 13.19 for ICA and sparse PCA (a lower number indicates a sparser weights of the SVM model), whereas it ranged between 13.33 and 14.51 for PCA and factor analysis. We thus found that activity maps can be described more correctly (according to model performance metrics) and more effectively (according to a model sparsity metric) by the learned network sets based on ICA and sparse PCA compared to components obtained from PCA and factor analysis. More generally, the observation of many zeros among the averaged model weights suggests that the “true” decomposition of task activity maps is a linear combination of few network components. Indeed, 18 diverse cognitive tasks could be very well distinguished solely based on 40 loadings of large-scale networks. The approach has been validated using four different decomposition methods in both HCP [50] and ARCHI [51] datasets. In sum, using task-derived network dictionaries (from an identical dataset), the extraction of and projection into network sets from ICA and sparse PCA achieved the highest prediction accuracies, precision and recall scores, as well as the most parsimonious model parameters. ICA and sparse PCA hence identified hidden networks underlying task settings that allowed the most efficient quantification of distinctive neural activity aspects.

After validating the proportional implication of large-scale networks as a salient property of task activity, we aimed at the interpretability of the network co-occurrence models from task maps (without any recourse to rest maps). The 18-task classification problem was solved by l2-penalized SVM of the selected k most important network loadings for each task (k = 40, 20, 10, 5, and 1) (Fig 3). As an exception to the generally employed l1-regularization of the SVM models, l2 penalization is used at this point because variable selection is already induced by the preliminary ANOVA-based selection (cf. methods section). The k best predictors for each task were inferred by classical univariate ANOVA tests for the network features that explain most variance between each task and the respective other tasks (Fig 4). Importantly, the determined single most important ICA or sparse PCA network loading per task yielded classification scores between 83% (ICA, standard deviation [SD] computed across participant-wise data folds in cross-validation scheme: 1.3) and 46% (Sparse PCA, across-fold SD = 1.7) for HCP as well as between 72% (ICA, SD = 3.0) and 38% (sparse PCA, SD = 4.7) for ARCHI. Increasing the number of k discriminative networks per task rapidly saturated the predictive accuracy. In both datasets, the classification accuracy was virtually identical when knowing all 40 or only the 20 most distinctive network loadings, which in turn was comparable to knowing only 10 and 5 network loadings. In sum, when assuming existence of 40 major networks, experimentally evoked neural activity patterns can be well discriminated by the relative implication of five task-activity-derived large-scale networks per experiment.

We then inspected the learned classification models based on task-derived network dictionaries as to whether they were *fit for purpose* [52]. The fitted model weights were unboxed by back-projection into whole-brain space (cf. methods section). It was thus quantified to what extent the winning explicit models capture genuine properties of fMRI task activity. This provided a real-world face-validity criterion to disambiguate whether task-immanent aspects of neural activity or arbitrary discriminative aspects (e.g., structured noise, participant/scanner-related idiosyncracies) explain the discriminative performance of a statistical model (Fig 5). Whole-brain activity maps were thus generated from the network co-occurrence models for each task (S6 and S7 Figs). The model-derived activity maps were then Pearson correlated with the mean first-level activity maps as a measure of recovery performance. For ICA decomposition, the mean linear correlation across 18 tasks reached r = 0.81 (HCP, standard deviation computed across tasks = 0.20) and r = 0.88 (ARCHI, SD = 0.07). For sparse PCA decomposition, in turn, mean correlations reached r = 0.69 (HCP, SD = 0.21) and r = 0.70 (ARCHI, SD = 0.15). As a side note, these findings were confirmed against the negative tests by pseudo-network priors derived from decomposition of Gaussian noise (i.e., 1000 noise maps with smoothed random activations). The corresponding correlation analyses between first-level task maps and model-derived task maps ranged between r = 0.32 (across-task SD = 0.07) and r = 0.25 (SD = 0.06). In sum, fMRI-task-activity derived neurobiological networks allowed significantly better reconstruction into the original activity space of >60.000 voxels than a dictionary of Gaussian random network templates. As an alternative to network co-occurrence models, whole-brain task activity was also captured by region co-occurrence models (Figs 5, S9 and S10). Capturing task activity in local cluster units, rather than distributed network units, was much less successful as clearly indicated by the acid test of reconstructing task-specific whole-brain activity from a few numbers alone (Figs 5, S9 and S10). Indeed, building a feature space that summarizes neural activity based on a ward cluster segregation of gray matter (out-of-sample accuracy 85% for HCP and 90% for ARCHI), the mean linear correlation between average class images and class reconstruction across 18 tasks reached only r = 0.11 (HCP, across-task SD = 0.12) and r = 0.26 (ARCHI, SD = 0.13). Summarizing neural activiy in k-means clusters (out-of-sample accuracy 86% for HCP and 89% for ARCHI), the mean linear correlation reached only r = 0.32 (HCP, across-task SD = 0.18) and r = 0.27 (ARCHI, across-task SD = 0.16). Finally, the *network*- and *region*-based reconstruction performances were compared to fitting the same l1-penalized support vector machine models on all 61,472 gray-matter *voxels* without the initial feature-learning step, that is, without recourse to global spatiotemporal components or local voxel clusters. Neurobiologically uninformed support vector machines applied to the raw voxel features (89% out-of-sample performance for HCP and 93% for ARCHI), otherwise identical to above classifier training by multi-class, one-versus-rest, and leave-one-participant-out design, achieved correlations between z-scored class average maps and z-scored model weights of r = 0.24 (across-task SD = 0.04) for HCP and r = 0.11 (across-task SD = 0.05) for ARCHI. Consequently, cluster-based and voxel-based multivariate predictive models were less successful in capturing the original task activity patterns than network co-occurrence models.

### Applying network co-occurrence modeling to test hypotheses: Task-rest correspondence

After evaluating network co-occurrence models, they were put into practice by testing explicit hypotheses about the difference between functional brain architecture of the human brain during tasks and at rest. In contrast to the above results, these experiments also performed regions/network discovery step and region/network-based task classification step based on non-identical data: HCP task maps, ARCHI task maps, and rest maps (Figs 6–8).

We formally tested whether given task activity patterns can be accounted for by network sets obtained from a diverging task battery and from the task-free resting brain (Figs 8, S11 and S12). To this end, recovery performance was compared between network sets learned from non-identical task data and from rest data. The rationale is that if functional brain architecture during task and at rest is similarly rich in variation patterns as measured by fMRI, then a rest-derived coordinate system should be able to effectively encode and reconstruct task activity maps as expressions on its 40 axes with little loss of information. How much information on the actual 3D activation patterns is lost by information compression in a network space is measured by the recovery performance. Learning network co-occurrence models from a non-identical task battery (i.e., networks from HCP task data and classification in ARCHI task data, or vice versa) yielded recovery performances between r = 0.50 (across-task SD = 0.17) and r = 0.43 (SD = 0.17) across two decompositions and datasets. Importantly, learning network co-occurrences models from task-unrelated resting-state correlations yielded very similar recovery performances between r = 0.51 (SD = 0.15) and r = 0.46 (SD = 0.11). Assessed by independent t-tests, recovery performance based on network sets from different task data was in no instance significantly better than recovery from networks discovered in task-unrelated rest data. This suggests that the network ecosystem of the mind-wandering brain as measured with fMRI scanning is recruited in a characteristic fashion in response to environmental challenges. The application of network co-occurrence models to the task-rest correspondence thus indicates that the directions of variation observed in the human brain at rest are sufficiently rich to explain the directions of variation observed in the human brain during task performance.

## Discussion

We confirm the central hypothesis that fMRI brain activity can be formally assessed in sets of macroscopical network units in a goal-direct task and an idlying rest mindset. The proposed statistical approach effectively exploits the recently increasing neurobiological evidence that functional coupling *between canonical brain networks* is more context-sensitive than functional coupling *within a same network* [15–17].

Two large datasets allowed for direct comparison of 36 diverse experimental tasks. A large proportion of activity patterns evoked by experimental tasks is shown to reflect combinations of stereotypical network patterns. Despite their diverging statistical assumptions (e.g., orthogonality, sparsity, independence, and heteroscedasticity), all used decomposition methods allowed for generative network models that can express neural activity maps as combinations of spatiotemporal activity components. We thus computed neurobiologically interpretable stratifications of large-scale network recruitment specific to cognitive tasks without relying on assumptions from cognitive theory cf. [98]. We validated decomposition and reconstruction of network ensembles in two large task fMRI datasets, each aimed at capturing human cognition comprehensively. Statistically, being able to create synthetic, never observed activity maps for each task is an important property of "generative models" [55]. Although the task-specific network implications are not directly observable using fMRI techniques, we demonstrate that the underlying organization can be numerically approximated in order to test explicit hypotheses.

Functional coupling between brain regions and coupling changes between task and rest has been investigated in several previous studies (e.g., [3, 4, 6, 56, 57]). *The novelty of the present approach consists in direct modeling of task activity by combinations of canonical brain networks and formal inference on changed neural activity of distinct networks*. *The involvement of major brain network ensembles in a given psychological task can be explicitly quantified at once and used for model-based prediction in independent or future neuroimaging studies*. The term "network co-occurrence" thus refers to the signature of network involvements specific to a given psychological task.

Up to now, the relationship between brain activity during task and rest has predominantly been addressed by three neuroscientific tools [2]: i) targeted neuroimaging experiments, ii) seed-based analysis of resting-state correlations, and iii) independent component analysis. How much experimental neuroimaging studies can contribute to research on task-rest correspondence is open to question. A paradox is introduced when attempting to measure *unconstrained* mind-wandering by imposing this behavior on the participants as *the task to follow* while lying in a brain imaging scanner cf. [58, 59, 60]. Additionally, each neuroimaging study can tap on only a small subset of possible experimental tasks. This precludes synoptic assessment of human cognition as a whole. Seed-based connectivity investigations, in turn, depend on the assumption that the seed-region definition successfully captures an underlying biological structure [61]. Seed correlation patterns also typically correspond to mixtures of overlapping canonical networks and are significance-tested in voxel space, which is statistically intractable and orthogonal to network interpretations [62, 108]. Moreover, ICA decomposes neuroimaging activations into a set of maximally statistically independent topographical maps in an *exploratory*, *hypothesis-free* fashion [46]. There is no obvious way to perform formal statistical *inference* on the implication of the ensuing network maps during psychological experiments [44, 47]. Although the number of such independent components is unknown [99–100], conventionally only one or two choices are explored [6, 7]. In sum, previous neuroimaging studies on task-rest correspondence analyzed and interpreted results in the neurobiologically invalid voxel space [108]. Additionally, the results were rarely assessed by inferential rejection of a null hypothesis or supervised comparison against some ground truth. Therefore, *network notions have so far hardly been formally tested for generalization to the population-level*.

It is an important limitation of the presented statistical framework that it ignores local within-network processes. The over-simplified quantification of brain activity at the network-level cannot account for relevant brain signal differences in circumscribed brain regions. Network co-occurrence modeling is hence insensitive to activity variance due to less distributed subnetwork effects underlying psychological tasks [101–102, 104, 107]. Findings that are likely to be neglected by the presented methodological approach include, for instance, task differences in the fusiform gyrus and the amygdala. Neural activity changes in the fusiform gyrus have repeatedly been shown to relate to information processing of human faces, especially stable facial characteristics like symmetry and scars [105]. The face-responsive fusiform gyrus recruitment typically localizes to its middle portion and does not co-occur with a notorious set of partner regions. Amygdala activity, in turn, responds reliably to environmental information that carries emotional, social, or motivational value, generally stimuli of evolutionary salience [106]. Similar to the fusiform gyrus, task response in the amgdala is not necessarily accompanied by neural activity changes in other parts of the limbic system or other canonical network. Therefore, facial and emotional processing subserved by local fine-grained activity changes in the fusiform gyrus and amygdala is probably not optimally characterized by network co-occurrence modeling. However, matrix factorization methods, such as ICA, PCA, sparse PCA, and FA, have been observed to decompose brain network patterns up to single-region nodes e.g., [6, 103]. Future research might therefore evaluate to what extent the identical statistical approach scales to the quantification of task effects subserved by local specialized brain regions after matrix factorization up to single-region patterns.

In the present study, the reconfiguration of large-scale networks was identified as a characteristic property of fMRI activity during experimental tasks. Multivariate decomposition of neural activity into 40 major networks allowed distinguishing 18 typical tasks in up to 90% (HCP dataset) and 93% (ARCHI dataset) of unseen activity maps. Selecting only the loading of the single most distinctive of 40 total networks for each task, classification still scored at up to 83% (HCP dataset) and 72% (ARCHI dataset). In particular, ICA and sparse PCA appeared most successful in searching the space of plausible network co-occurrence architectures. Neurobiological pertinence was suggested by overall best task classification scores, highest parsimony in model parameters, and model-derived recovery of most realistic task activity maps. This suggests that the underlying statistical assumptions of these models reverse-engineer critical properties of brain network constellation. In this way, present results would favor large-scale patterns to parsimoniously distribute in space (reflected by sparse PCA models) and vary independently (reflected by ICA models), whereas orthogonality (reflected by PCA) and different variances (reflected by factor analysis) seem less important. Notably, attempting the reconstruction of statistically coherent source signals operates with a network definition that is not strictly neurobiological in nature [63]. Nevertheless, a large proportion of task activity can be explained by a combination of the brain’s main functional networks. This study might be the first to carefully quantify the often held view that contrast approaches of experimental fMRI studies do not necessarily elicit idiosyncratic activity patterns, but mainly a recombination of the same underlying networks cf. [44, 64, 65–67]. This is true in the sense that *rest network dictionaries have been shown to reflect sufficiently rich directions of variation to encode and reconstruct whole-brain task images with minimal information loss*. It follows that a single major brain network may not be task-specific (e.g., the "language network", the "empathy network") but the entire activity pattern of the various brain networks may be a distinctive correlate underlying each cognitive process. Indeed, an interplay of limited entities generates an infinity of different observations in numerous natural systems [68], formalized in General System Theory [69]. This contention lends itself to diverse set of neuroscientific questions that currrent lack a pertinent methodological access.

Finally, we have demonstrated the usefulness of constructing and comparing network co-occurrence models to test specific hypotheses about network architecture in fMRI data. We tested between what one can summarize as “dichotomy” and “manifold” hypotheses on the neuroarchitectural difference between task behaviors and the idling brain [70]. While the dichotomic view revolves around functional antagonism between so-called “task-positive” and “task-negative” neural networks, the “manifold” view advocates a fluctuating equilibrium of functionally distinct large-scale networks that is perturbed at task onset. In the present work, we formally tested dichotomy against manifold hypotheses by learning independent network co-occurrence architectures based on non-identical task and rest data. Across four diverging decomposition methods, recovery performance from task-derived network dictionaries was in no instance significantly better than rest-derived network dictionaries. This indicator of equal network repertoires across task and rest brain dynamics favors the manifold perspective on task-rest correspondence. Consistent with Smith and colleagues [6], explicit network co-occurrence models challenge the frequently embraced separation into “extrinsic” and “intrinsic” brain systems [71]. Our approach further reconciles the previous contention that activation patterns in certain brain areas may be richer during task than at rest [4]. The same set of canonical networks might give rise to task-evoked network compositions that are however seldomly observed at rest. In sum, the conducted network co-occurrence experiment suggests that task-constrained onset of cognitive processes modulates an identical repertoire of large-scale brain networks.

### Conclusion

Only recently, systems neuroscience has transitioned the interpretational focus from regional segregation to network integration [18, 28, 37, 72]. Many neuroimaging studies then performed ICA without relation to human cognitive processes or conducted resting-state correlations yielding unknown mixtures of constituant brain networks. The present study may be the first to quantify the composition of large-scale network recruitments during defined psychological tasks and to thus qualify a meaningful mechanism underlying cognitive neuroscience experiments. Task-specific network compositions might thus intimately relate to psychological notions of mental operations, although fine-grained local effects are ignored. This organizational principle of the human brain might extend to other species given existence of large-scale networks in monkeys [73] and rats [74]. Moreover, clinical research has corroborated default-mode network dysfunction in various psychiatric and neurological disorders [75, 76]. As a tempting alternative hypothesis for future research, not disturbance in the default-mode network itself but its relation to other canonical networks might be specific to brain disorders. Ultimately, the present investigation exposes network co-occurrence architecture as an important neural mechanism that complements regional brain responses in maintaining human cognition.

## Materials and Methods

### Data

#### Dataset: HCP task data

The first of two task batteries was drawn from the Human Connectome Project (HCP; www.humanconnectomeproject.org). HCP is an international long-term project dedicated to network exploration [77]. 500 HCP participants (2 removed for quality reasons) were without psychiatric or neurological history. Informed consent was obtained from all participants by the Washington University in St. Louis institutional review board. The ethics approval for using the HCP data was obtained from the local ethics committee of the Heinrich-Heine University Düsseldorf, Germany. Network discovery is facilitated by probing experimental task paradigms that are known to tap on well-characterized neural networks. This was achieved by selecting tasks that feature known suitability and reliability across participants [50]. The HCP tasks (Table 1) were chosen by the external advisory board, consortium members, and HCP team from the National Institut of Mental Health. Over two image acquisition sessions, mostly block-design, but also event-related, paradigms were administered on 1) working memory/cognitive control processing, 2) incentive processing, 3) visual and somatosensory-motor processing, 4) semantic and phonological language processing, 5) social cognition, 6) relational processing, and 7) emotional processing. All data were acquired on the same Siemens Skyra 3T scanner at Washington University. Whole-brain EPI acquisitions were acquired with a 32 channel head coil (TR = 720ms, TE = 33.1ms, flip angle = 52, BW = 2290Hz/Px, in-plane FOV = 28 × 18cm, 72 slices, 2.0mm isotropic voxels). One task was run with right-to-left and one with left-to-right phase encoding. We profited from the HCP minimally preprocessed pipeline [78]. This includes gradient unwarping, motion correction, fieldmap-based EPI distortion correction, brain-boundary-based registration of EPI to structural scan, non-linear (FNIRT) registration into Montreal Neurological Institute (MNI) space, and grand-mean intensity normalization. The preprocessed maps were spatially smoothed by a Gaussian kernel of 4mm (FWHM). A general linear model (GLM) was implemented by FILM from the FSL suite with model regressors from convolution with a canonical hemodynamic response function and from temporal derivatives. It is important to note that HCP tasks were conceived to modulate activation in a maximum of different brain regions and neural systems. Indeed, excellent brain coverage was indicated by the summing the maps of whether or not a voxel showed a z-value bigger than 1.96 per task across participants [50]. Note that we statistically analyzed the GLM-derived participant-level z-score maps in MNI space.

**Table 1:** HCP task descriptions.

Cognitive Task	Stimulus material	Instruction to participants
1 Reward	Card game	Guess the number of mystery card for gain/loss of money
2 Punish	Card game	Guess the number of mystery card for gain/loss of money
3 Shapes	Shape pictures	Decide which of two shapes matches another shape geometry-wise
4 Faces	Face pictures	Decide which of two faces matches another face emotion-wise
5 Random	Videos with objects	Decide whether the objects act randomly or intentionally
6 Theory of mind	Videos with objects	Decide whether the objects act randomly or intentionally
7 Mathematics	Spoken numbers	Complete addition and subtraction problems
8 Language	Auditory stories	Choose answer about the topic of the story
9 Tongue movement	Visual cues	Move tongue
10 Food movement	Visual cues	Squeezing of the left or right toe
11 Hand movement	Visual cues	Tapping of the left or right finger
12 Matching	Pictures	Decide whether two objects match in shape or texture
13 Relations	Pictures	Decide whether object pairs differ both along either shape or texture
14 View Bodies	Pictures	Passive watching
15 View Faces	Pictures	Passive watching
16 View Places	Pictures	Passive watching
17 View Tools	Pictures	Passive watching
18 Two-Back Memory Task	Pictures	Indicate whether current stimulus is the same as two items earlier

Further details can be obtained from the original task dataset descriptions [50].

#### Dataset: ARCHI task data

The second task battery was drawn from the ARCHI dataset [51]. Importantly, it was conceived and acquired with the same goal for neural networks shared by a maximum of participants as the HCP task dataset. The diverse experimental tasks (Table 2) capture the cerebral basis of auditory and visual perception, motor action, reading, language comprehension and mental calculation. The ethics approval was obtained from the regional ethical committee (Hôpital de Bicêtre, France). 81 right-handed healthy participants (35 males and 46 females, 24 +/- 4.1 years, three not included in present analyses due to incomplete data) without psychiatric or neurological history participated in four fMRI sessions acquired under different experimental paradigms. The functional maps were warped into the MNI space and resampled at 3mm resolution. Task maps were computed using a GLM based on the specified task timing information, canonical hemodynamic response function, high-pass filtering, and auto-regressive noise model. The tasks included 1) a general paradigm that probes basic functions, such as pressing a button with the left or right hand, viewing horizontal and vertical checkerboards, reading and listening to short sentences, and mental computations (subtractions), 2) a social paradigm that includes covertly making some inferences on short stories that involve false beliefs or not, viewing objects moving with or without a putative intention, listening to speech and non-speech sounds, 3) a spatial mapping paradigm that includes perfoming ocular saccades, grasping, and orientation judgements on objects (the two different tasks were actually made on the same visual stimuli in order to characterize grasping-specific activity), as well as judging whether a hand photograph corresponds to the left or right hand or was displaying the front or back, 4) an emotional paradigm that include facial judgments of gender, trustworthiness, and expression based on face photographs or photographs reduced to the eyes. While the first experimental paradigm is fast event-related design, the two others are made of small blocks of 5 to 7 seconds for each condition. The duration of the acquisitions was 1) 307s, 2) 489s, 3) 516s, and 4) 436s. Visual stimuli were displayed in four 250-ms epochs, separated by 100ms intervals (i.e., 1.3s in total). Auditory stimuli were drawn from a recorded male voice (i.e., a total of 1.6s for motor instructions, 1.2–1.7s for sentences, and 1.2–1.3s for subtraction). The auditory or visual stimuli were shown to the participants for passive viewing or button response in event-related paradigms. Post-scan questions verified that the experimental tasks were understood and followed correctly. Based on these data, the following functional contrasts were computed and used in the following inference: 1) basic paradigm, left versus right hand button pressure, horizontal versus vertical checkerboard, auditory versus visual instructions, computation versus simple reading, motor tasks versus language, and math tasks, 2) social paradigm: speech versus non-speech sounds, false belief versus mechanistic inference after visual or auditory presentation, interacting versus non-interacting figures in the movie clip, 3) spatial mapping: grasping versus orientation judgement, left/right hand versus hand side, effect of ocular saccades, 4) emotional paradigm: gender judgement versus no task on face image and expression, truthworthiness versus gender judgement and baseline. Whole-brain EPI data were acquired with the same Siemens Trio with a 32 channel head coil (TR = 2400ms, TE = 30ms, flip angle = 60, in-plane FOV = 19.2 × 19.2cm, 40 slices, 3.0mm isotropic voxels). A posterior-anterior phase encoding scheme was used for all images. Standard preprocessing was performed with Nipype (Gorgolewski et al., 2011), including slice timing, motion correction, alignment, and spatial normalization. Activation maps were spatially smoothed by a Gaussian kernel of 5mm (FWHM). Note that we statistically analyzed the GLM-derived participant-level z-score maps in MNI space, analogous to the HCP task data.

**Table 2:** ARCHI task descriptions.

Cognitive Task	Stimulus material	Instruction to participants
1 Face discrimination	Visual face pictures	Covert response to gender
2 Face discrimination	Visual face pictures	Covert response to trustworthiness
3 False belief	Auditory	Covert answer to "Why?"
4 False belief	Moving triangles	Passive viewing
5 Object grasping	Pictures of everyday objects	Move hand as if to use that object
6 Object orientation	Pictures of everyday objects	Use fingers to indicate whether object is inclined to the right or left
7 Rotation hand	Photos of hands at different angles	Covert response whether hand back or palm is shown
8 Rotation side	Photos of hands at different angles	Covert response whether left or right hand is shown
9 Motor-cognitive	Abstract cues	Motor action according to cue
10 Hand movement	Auditory sentence	Press the left button 3 times
11 Hand movement	Auditory sentence	Press the right button 3 times
12 Saccades	Crosshair suddenly shifts from center to screen periphery	Keep eyes on crosshair without head movement
13 Speech	Auditory sentences	Passive listening
14 Reading	Written stories	Passive appraisal
15 Story comprehension	Auditory short stories	Covert answer to questions
16 Computation	Auditory sentences	Subtract numbers and keep result in mind
17 Auditory	Series of sounds	Passive listening
18 Video	Flipping abstract objects	Passive viewing

Further details can be obtained from the original task dataset descriptions [51].

#### Dataset: Rest data

These two task datasets were complemented by HCP resting-state acquisitions of brain activity in the absence of experimental paradigms. In line with the goal of the present study, acquisition of these data was specifically aimed at the study of task-rest correspondence [77]. The rest maps were acquired in two imaging sessions. Each participant contributed four time-series (2 sessions each for left-to-right and right-to-left phase encoding) with 1,200 maps of multiband, gradient-echo planar imaging acquired during a period of 15min (TR = 720 ms, TE = 33.1 ms, flip angle = 52, FOV = 280 × 180mm, and 2.0mm isotropic voxels). Besides run duration, the task acquisitions were identical to the resting-state fMRI acquisitions for maximal compatibility between task and rest data. The unusually low TR allowed for effective increase in the signal-to-noise ratio and removal of physiological confounds. During map acquisition, participants fixated on a bright crosshair on dark background. We drew on “minimally preprocessed” rest data from 25 randomly selected healthy participants. Every participant who contributed scans to the present rest dataset was included with one left-right and one right-left phase encoding session. Importantly, PCA was applied to each set of 1,200 rest maps for denoising and dimensionality reduction into 20 main modes of variation [79]. That is, the 40 reduced rest maps for each of 25 randomly selected participants constituted the present rest dataset of 1000 concatenated, noise-cleaned rest activity maps.

In sum, the HCP task data incorporated 8650 first-level activity maps from 18 diverse paradigms administered to 498 participants, while the ARCHI task data incorporated 1404 first-level activity maps from 18 diverse paradigms administered to 78 participants. The rest data, in turn, incorporated 1000 activity maps drawn from different scanning sessions in 25 participants. The maps from all three datasets were downsampled to a common 53 × 63 × 46 space of 3mm isotropic voxels. The resampled activity maps were masked by a gray-matter probability of at least 10% according to tissue maps from the Intertional Consortium of Brain Mapping (ICBM). Each of the task and rest maps was hence represented by 61,472 voxels with z-values in gray matter.

### Workflow

Data folding and model selection were performed in the following fashion. In the first step, one half of the task datasets (i.e., HCP and ARCHI) and the entire rest dataset were used for *unsupervised* (i.e., label-independent) discovery of latent structure by matrix decomposition and clustering methods. The components of variation identified in the data allowed for feature engineering from biological structures. In the second step, we applied *supervised* (i.e., label-dependent) classification algorithms to the other half of the activity maps to predict cognitive tasks. The winning models for classifying 18 cognitive tasks were compared by cross-validation. This inferential statistical framework is the gold standard to obtain an unbiased estimate of how well a trained classifier generalizes beyond the data samples at hand [80, 81]. The previously unseen half of the task data (i.e., 4325 maps from HCP and 702 maps from ARCHI] were split into as many data folds as participants (i.e., 498 for HCP and 78 for ARCHI]. In each fold, all task maps of a given participant were left out as the test set for assessment of out-of-sample performance, while the task maps from the remaining participants served as the training set for model estimation. This *leave-one-participant-out cross-validation* scheme ensured model fitting of task effects, rather than interindividual differences. In the third and last step, the averaged winning models were back-projected into realistic task activity maps as face validity for the reduced representation of task activity patterns.

#### 1. Unsupervised analysis layer

The first step uncovered hidden structure in large quantities of neural activity maps. To this end, each dataset (i.e., first half of task datasets and entirety of rest dataset] was decomposed into the 40 modes of variation. A large set of 40 “network" archetypes should ensure more parsimonious representation of the unknown target function, despite increased problem difficulty due to higher variance. Latent large-scale networks were computed by maximizing the intra-network homogeneity in neural activity and maximizing the between-network heterogeneity. We selected four different decomposition methods that are in frequent general use.

*Decomposition into brain networks*. Four different network decomposition techniques were applied to the neural activity maps in order to test whether the neuroscientific findings generalize across diverging methodological choices. First, *independent component analysis* (ICA] was used to unmix the multivariate BOLD signals in the activity maps into separate spatial components by minimizing their mutual information [82]. This iterative algorithm for blind source separation was realized by a parallel FastICA implementation (200 maximum iterations, per-iteration tolerance of 0.0001, initialized by a random mixing matrix, whitening). In neuroimaging research, ICA is frequently employed to separate out stable, statistically independent spatial patterns with identical time courses for the nodes of each extracted network [46]. Second, *principal component analysis* (PCA) was used to capture the main directions of variation in the activity maps [83]. This non-parametric, rotation-invariant method assumes orthogonal components to remove second-order dependencies (i.e., covariance) between the voxels by a change of basis [48]. An implementation of incremental PCA [84] performed batch-by-batch decomposition for low-rank approximation with efficient memory usage (batch size = 100 activity maps, whitening). Third, *sparse PCA* was used to separate the activity maps into network components with few regions [85], which scales well to large datasets. This decomposition method reformulates a PCA-like modeling goal as a regression-type optimization problem constrained by l1-penalty terms. An implementation without orthogonality assumptions yielded spatially less distributed components to explain the variation in the data (1000 maximum iterations, per-iteration tolerance of 1 * 10–8, sparsity alpha = 1, ridge-shrinkage at 0.01, Lasso solution computed with coordinate descent) [86]. Fourth, *factor analysis* (FA) generalizes (probabilistic) PCA by a heteroscedastic noise model [55]. Similar to PCA, FA performs a rotation-invariant low-rank approximation by identifying the latent space of variation (1000 maximum iterations, per-iteration tolerance of 0.01, 3 iterations in power method, randomized singular vector decomposition).

Note that all four decomposition methods implicitly assume large variance to be indicative of impor- tant structure. They reflect different ways to model sets of partially-overlapping major brain networks without access to any task information. All decompositions are soft in that each voxel with its BOLD signals can be part of the support in more than one network component. For most (ICA, PCA, FA) but not all (sparse PCA) decompositions, the ensuing components are linear combinations of *all* the original voxel signals.

*Clustering into brain regions*. The decompositions into spatially overlapping network components were evaluated against clustering of the same data into non-overlapping regional components. That is, the similarity between voxels in neural activity changes across maps was indicative of coherent regional compartments, rather than distributed spatiotemporal networks. On the one hand, Ward clustering is a bottom-up hierarchical clustering approach [87]. A spatial constraint ensured that only neighboring voxels were incorporated into regional components of similar variation in BOLD signals [88]. On the other hand, K-means is a popular top-down clustering approach [89]. It divided the voxels into a preselected number of k regional components of variation without a spatial constraint [90].

Note that the clustering methods thus yielded a regional scaffold of functional brain architecture with emphasis on functional specialization by segregation into disjoint region units. Yet, the decomposition methods yielded a spatially distributed scaffold with emphasis on functional integration by separation into overlapping network units. Please note however that clustering methods impose stricter constraints on the estimation of relevant structure, including the flat partition of the voxel space and the compulsory assignment of each voxel to one cluster [45, 88, 91]. The rationale was that four network co-occurrence models and two region co-occurrence models embodied quantitative proxies of functional integration versus functional specialization concepts of the human brain (cf. introduction section).

#### 2. Supervised analysis layer

The extracted hidden components were subsequently used to reduce each task activity map to a much smaller number of component loadings. 61,472 voxels from gray-matter voxels of each map were thus condensed into 40 component loadings that quantify task-related network co-occurrence or region co-occurrence involvements. For network co-occurrence models, the presence of large-scale networks in every task map was determined by ridge regression based on a component design matrix (regularization alpha parameter = 0.001, using Cholesky solver). 40 component loadings were thus computed by projection of each task map onto the 40 network components. Note that the proposed approach is thus related to dual regression analysis [92]. For region co-occurrence models, 40 component loadings were computed by the cluster-wise mean of the BOLD signal. Task-related brain activity was thus summarized by brain regions of homogeneous activity changes across tasks. In both these approaches, the feature space for supervised classification had the form: #samples (i.e., 4325 HCP task maps or 702 ARCHI task maps) × #features [40 component loadings). Linear support vector machines (SVM) were used to approximate the unknown target function that perfectly classifies the 18 cognitive tasks. This discriminative maximum-margin classifier was chosen for its simplicity and very good out-of-sample performance [93, 94]. We preprocessed the feature space by normalization and mean-centering of each individual voxel due to sensitivity of this procedure to scaling effects [52].

In particular, linear-SVM classification was performed with regularization by l1- and l2-penalty terms. Both shrinkage methods were used to search for parsimonious, more interpretable models [80]. First, l1-regularized SVM analyses (used in most analyses) conducted i) feature selection (i.e., select only relevant components) and ii) model estimation (i.e., determine what combination of components best disentangles the cognitive tasks) in an identical process. This approach introduced a maximum of zero-ed SVM weights for automatic determination of optimal model complexity. Note that prior dimensionality reduction (i.e., decomposition or clustering) yielded a set of new features that should exhibit much less multi-collinearity than those of the initial voxel space (i.e., task and rest activity maps). From a neurobiological perspective, this approach acknowledged the assumption that smaller groups of, but not all, network components should be characteristic for the observed activity pattern of the various tasks. We performed hyperparameter tuning by a grid-search of the SVM hyperparameter “C” (7 steps between 10–3 and 103 on a logarithmic scale) and averaged the fold-wise model parameters (i.e., bagging; 80). The amount of sparsity (induced by l1-constrained SVMs) was thus adapted to best predicting cognitive tasks. The collapsed SVM classification models thus represented the average hypothesis from that dataset with reduced variance but essentially unchanged bias. Second, the l2-regularized SVM analyses (only used by analyses described in next passage) were used for the tendency to shrink the model parameters i) towards zero and ii) towards each other. In so doing, we shrunk all directions of variation. Comparing to l1-regularized, no implicit feature selection is performed as the ensuing parameters are typically small but non-zero. That is, both l1 and l2 penalization perform variable shrinkage, yet l2-constrained SVM does not attempt to improve sparsity and domain interpretability by introducing zero coefficients.

In one subanalysis, ANOVA was used for explicit univariate component selection of the k most important (i.e., 1, 5, 10, and 20) component loadings for each given task comparing to other tasks. That is, fitting the multivariate classifier was preceded by a univariate feature-selection procedure on the training data in each cross-validation fold. In particular, the feature space (i.e., 40 component involvements) was reduced to the k component loadings most relevant for each task. As we had already selected variables in this feature space, it was fed to l2-penalized, rather than l1-penalized, SVM to delineate their task-specific contribution. From a neurobiological perspective, this approach acknowledged the assumption that the balanced contribution of few large-scale networks should be sufficient to disentangle cognitive tasks. Note that all other reported results were based on the full set of 40 network or region loadings without ANOVA-based subselection.

#### 3. Validation layer

We finally evaluated whether the obtained explicit models capture genuine properties of fMRI task activity. Complementing sparsity and predictive performance, this provides a face-validity criterion to disambiguate whether task-immanent aspects of neural activity or arbitrary discriminative aspects (e.g., structured noise, participant/scanner-related idiosyncracies) explain the models’ success. To this end, artificial maps were generated from the explicit network co-occurrence or region co-occurrence models of each task. The 40 features describing large-scale network or region implications in a given task were transformed back into the original gray-matter voxel space with 61,472 voxels. In network decomposition, the product was computed between the SVM weights and the support of each component. In region clustering, the mean activity of each region was projected into each voxel of that region. Importantly, back-projection from an abstract, highly reduced space to the original task activity space should succeed as a function of the construct validity of each model. For each of 18 tasks, Pearson’s correlation between the model-derived activity map and the mean across first-level activity maps quantified the recovery performance. Consequently, the recovery performance indexed the neurobiological validity of quantitative network co-occurrence and region co-occurrence models.

### Software implementation

Python was selected as scientific computing engine. Capitalizing on its open-source ecosystem helps enhance replicability, reusability, and provenance tracking. Nipy [95] performed basic analyses of functional neuroimaging data (http://nipy.org/). Scikit-learn [96] provided efficient, unit-tested implementations of state-of-the-art statistical learning algorithms (http://scikit-learn.org). This general-purpose machine-learning library [97] was interfaced with the neuroimaging-specific nilearn library for high-dimensional neuroimaging datasets (http://github.com/nilearn/nilearn). 3D visualization of brain maps was performed using PySurfer (http://pysurfer.github.io/). All analysis scripts of the present study are readily accessible to the reader online (http://github.com/banilo/taskrest2016).

## Supporting Information

S1 Fig Sparse PCA network decomposition of HCP task maps and network-task assignment. 40 network components underlying 18 HCP tasks (left) have been discovered by sparse PCA (Comp1-40). The ensuing network loadings from the second half of the HCP task data were submitted to classification of the psychological tasks based on the implication of brain networks (l2-penalized support vector machines, multi-class, one-versus-rest). l2-contrained support vector machines were employed to choose the most discriminatory network variables by a classical univariate test in a discrete fashion rather than by soft variable selection based on l1 penalization (cf. methods section). This diagnostic analysis (right) revealed the most distinctive k = 1, 5, 10, and 20 network features (red cubes) for each experimental condition of the task battery (cf. Fig 3). The thus discretely selected network features per task were then fed into supervised multi-task classification as a feature space of activity-map-wise continuous activity values. The color intensity of the k cubes quantifies how often the corresponding brain network was selected as important for a task across cross-validation folds. This diagnostic test performed inference on a) the single most discriminative network for each task at k = 1, b) the network variables that get added step-by-step to the feature space of network implications with increasing k, and c) what network variables are unspecific (i.e., not selected) for a given task at k = 20. See Table 1 of the manuscript body for descriptions of task 1 to 18.
(PNG)

S2 Fig ICA network decomposition of HCP task maps and network-task assignment. 40 network components underlying 18 HCP tasks (left) have been discovered by ICA (Comp1-40). The ensuing network loadings from the second half of the HCP task data were submitted to classification of the psychological tasks based on the implication of brain networks (l2-penalized support vector machines, multi-class, one-versus-rest). l2-contrained support vector machines were employed to choose the most discriminatory network variables by a classical univariate test in a discrete fashion rather than by soft variable selection based on l1 penalization (cf. methods section). This diagnostic analysis (right) revealed the most distinctive k = 1, 5, 10, and 20 network features (red cubes) for each experimental condition of the task battery (cf. Fig 3). The thus discretely selected network features per task were then fed into supervised multi-task classification as a feature space of activity-map-wise continuous activity values. The color intensity of the k cubes quantifies how often the corresponding brain network was selected as important for a task across cross-validation folds. This diagnostic test performed inference on a) the single most discriminative network for each task at k = 1, b) the network variables that get added step-by-step to the feature space of network implications with increasing k, and c) what network variables are unspecific (i.e., not selected) for a given task at k = 20. See Table 1 of the manuscript body for descriptions of task 1 to 18.
(PNG)

S3 Fig ICA network decomposition of ARCHI task maps and network-task assignment. 40 network components underlying 18 ARCHI tasks (left) have been discovered by ICA (Comp1-40). The ensuing network loadings from the second half of the ARCHI task data were submitted to classification of the psychological tasks based on the implication of brain networks (l2-penalized support vector machines, multi-class, one-versus-rest). l2-contrained support vector machines were employed to choose the most discriminatory network variables by a classical univariate test in a discrete fashion rather than by soft variable selection based on l1 penalization (cf. methods section). This diagnostic analysis (right) revealed the most distinctive k = 1, 5, 10, and 20 network features (red cubes) for each experimental condition of the task battery (cf. Fig 3). The thus discretely selected network features per task were then fed into supervised multi-task classification as a feature space of activity-map-wise continuous activity values. The color intensity of the k cubes quantifies how often the corresponding brain network was selected as important for a task across cross-validation folds. This diagnostic test performed inference on a) the single most discriminative network for each task at k = 1, b) the network variables that get added step-by-step to the feature space of network implications with increasing k, and c) what network variables are unspecific (i.e., not selected) for a given task at k = 20. See Table 2 of the manuscript body for descriptions of task 1 to 18.
(PNG)

S4 Fig Sparse PCA network decomposition of rest maps. Sparse PCA decomposition was used to derive the 40 most important modes of variation (Comp1-40) in the rest data (cf. methods section). These parsimonious spatial patterns are depicted in coronal, sagittal, and axial slices rendered on the Colin MNI template. Combinations of this dictionary of overlapping major brain networks were used to explain task-evoked neural activity patterns.
(PNG)

S5 Fig ICA network decomposition of rest maps. ICA decomposition was used to derive the 40 most important modes of variation (Comp1-40) in the rest data (cf. methods section). These independent spatial patterns are depicted in coronal, sagittal, and axial slices rendered on the Colin MNI template. Combinations of this dictionary of overlapping major brain networks were used to explain task-evoked neural activity patterns.
(PNG)

S6 Fig Reconstruction of HCP tasks from different network co-occurrence models. Leftmost column: Average whole-brain activity from 18 experimental tasks of the HCP dataset. The voxel-wise mean was computed across the first-level contrast activity maps from 498 participants. Right columns: Four different network models were derived from the task activity during the 18 HCP tasks. First, four different network decompositions (i.e., ICA, sparse PCA, PCA, and factor analysis) were applied to the first half of the HCP task data. Second, the ensuing sets of 40 brain networks served as a basis for feature engineering to automatically learn distinguishing the 18 tasks in the second data half based on network loadings alone. Third, the quantitative models of task-specific network loadings allowed generating a synthetic whole-brain activity map for each experimental task. The correlation values r quantify the voxel-wise similarity between the reconstructed activity map and the average activity map for each task and network decomposition method. This measure of recovery performance indicates the information loss incurred when first expressing activity maps as 40 network loading values and then translating these values back into whole-brain space. The similarity between real HCP task maps (leftmost column) and synthetic model-derived task maps (right columns) indicates that 40 network loadings can well describe task-evoked neural activity patterns in the HCP task battery.
(JPG)

S7 Fig Reconstruction of ARCHI tasks from different network co-occurrence models. Leftmost column: Average whole-brain activity from 18 experimental tasks of the ARCHI dataset. The voxel-wise mean was computed across the first-level contrast activity maps from 78 participants. Right columns: Four different network models were derived from the task activity during the 18 ARCHI tasks. First, four different network decompositions (i.e., ICA, sparse PCA, PCA, and factor analysis) were applied to the first half of the HCP task data. Second, the ensuing sets of 40 brain networks served as a basis for feature engineering to automatically learn distinguishing the 18 tasks in the second data half based on network loadings alone. Third, the quantitative models of task-specific network loadings allowed generating a synthetic whole-brain activity map for each experimental task. The correlation values r quantify the voxel-wise similarity between the reconstructed activity map and the average activity map for each task and network decomposition method. This measure of recovery performance indicates the information loss incurred when first expressing activity maps as 40 network loading values and then translating these values back into whole-brain space. The similarity between real HCP task maps (leftmost column) and synthetic model-derived task maps (right columns) indicates that 40 network loadings can well describe task-evoked neural activity patterns in the ARCHI battery.
(JPG)

S8 Fig Region clustering of task activity. 40 clusters of homogeneous neural activity patterns across tasks and across participants. Spatially constrained ward and spatially unconstrained k-means clustering were applied to the first half of the task maps from the HCP and ARCHI dataset. Each cluster is depicted in a unique, arbitrary color. This grey-matter atlas of non-overlapping brain regions was used to explain task-evoked neural activity patterns.
(PNG)

S9 Fig Reconstruction of HCP tasks from different region co-occurrence models. Leftmost column: Average whole-brain activity from 18 experimental tasks of the HCP dataset. The voxel-wise mean was computed across the first-level contrast activity maps from 498 participants. Right columns: Two different region models were derived to explain the task activity during the 18 HCP tasks. First, two different clustering algorithms (i.e., ward and k-means clustering) were applied to the entirety of the HCP task data. Second, the ensuing sets of 40 coherent activation clusters served as a basis for feature engineering to automatically learn to distinguish the 18 tasks based on cluster loadings alone. Third, the quantitative models of task-specific cluster loadings allowed generating a synthetic whole-brain activity map for each experimental task. The correlation values r quantify the voxel-wise similarity between the reconstructed activity map and the average activity map for each task and region clustering method. This measure of recovery performance indicates the information loss incurred when first expressing activity maps as 40 region summary values and then translating these values back into whole-brain space. The frequently low similarity between real HCP task maps (leftmost column) and synthetic model-derived task maps (right columns) indicates that 40 cluster loadings might not well describe task-evoked neural activity patterns in the HCP battery.
(JPG)

S10 Fig Reconstruction of ARCHI tasks from different region co-occurrence models. Leftmost column: Average whole-brain activity from 18 experimental tasks of the ARCHI dataset. The voxel-wise mean was computed across the first-level contrast activity maps from 78 participants. Right columns: Two different region models were derived to explain the task activity during the 18 ARCHI tasks. First, two different clustering algorithms (i.e., ward and k-means clustering) were applied to the entirety of the ARCHI task data. Second, the ensuing sets of 40 coherent activation clusters served as a basis for feature engineering to automatically learn distinguishing the 18 tasks based on cluster loadings alone. Third, the quantitative models of task-specific cluster loadings allowed generating a synthetic whole-brain activity map for each experimental task. The correlation values r quantify the voxel-wise similarity between the reconstructed activity map and the average activity map for each task and region clustering method. This measure of recovery performance indicates the information loss incurred when first expressing activity maps as 40 region summary values and then translating these values back into whole-brain space. The frequently low similarity between real ARCHI task maps (leftmost column) and synthetic model-derived task maps (right columns) indicates that 40 cluster loadings might not well describe task-evoked neural activity patterns in the ARCHI battery.
(JPG)

S11 Fig Recovery performance of task activity patterns across decomposition and clusterings. Four different network co-occurrence models (upper and middle row) were computed based on decomposition based on sparse PCA, ICA, PCA, and factor analysis (FA). They capture functional brain architecture with emphasis on functional integration as opposed to regional specialization. Two different region co-occurrence models (lower row) were computed based on ward clustering (regions always spatially connected) and k-means clustering (no spatial constraint). They capture functional brain architecture with emphasis on regional specialization. The recovery performance of all 18 tasks (radial columns) is measured by the Pearson correlation r between the model-derived task activity maps and the average first-level task map. As a first conclusion, modeling task-specific neural activity appears to be more successful based on functional network units than on functional region units. Additionally, network and region dictionaries were derived from i) identical task-data as positive test (dark blue), ii) non-identical task-data (medium blue), iii) resting-state data (light blue), and iv) Gaussian noise as negative test (red). As a second conclusion, network dictionaries derived from non-identical task maps and rest maps are similarly successful in recovering whole-brain activity during divering experimental tasks. This was confirmed by univariate t-tests between the task-wise correlation values r (cf. results section).
(PNG)

S12 Fig Recovery performance of task activity patterns across tasks. Parallel coordinate plots that depict the recovery performance by one line for each of the 18 tasks in both datasets. The network dictionaries were derived from i) identical task-data (dark blue, AT = ARCHI task data, HT = HCP task data), ii) non-identical task-data (medium blue), iii) resting-state data (light blue, HR = HCP rest data), and iv) Gaussian noise (red, DN = data noise), analogous to S11 Fig. An important conclusion is that network dictionaries derived from non-identical task maps and rest maps are similarly successful in recovering whole-brain activity during divering experimental tasks. This was confirmed by univariate t-tests between the task-wise correlation values r (cf. results section).
(PNG)
