# Supplementary figures and images for: Formal Models of the Network Co-occurrence Underlying Mental Operations
Source: PLoS Comput Biol. 2016 Jun 16;12(6):e1004994. doi: 10.1371/journal.pcbi.1004994 (PMC4911040; doi:10.1371/journal.pcbi.1004994)

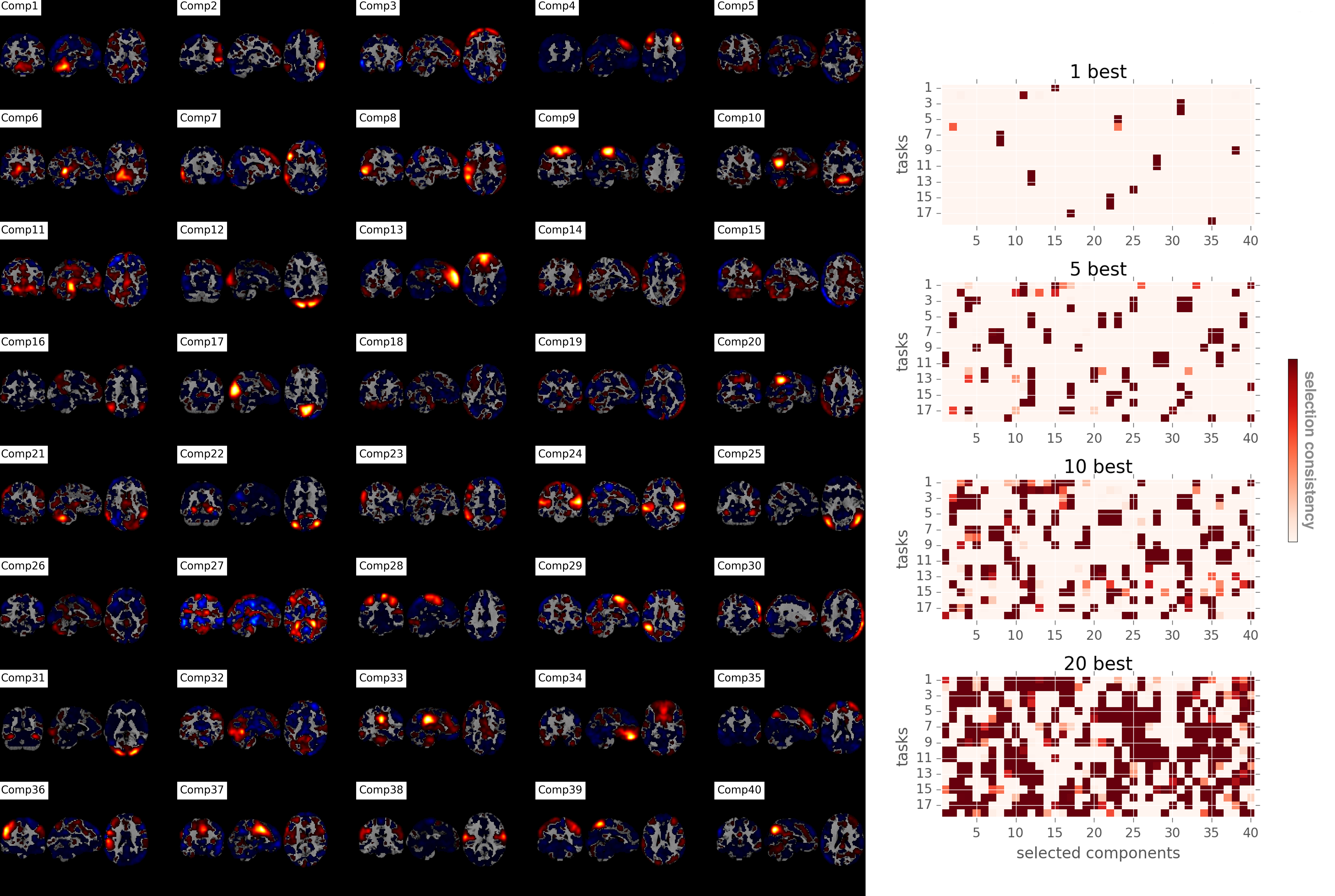

Supplement: S1 Fig — 40 network components underlying 18 HCP tasks (left) have been discovered by sparse PCA (Comp1-40). The ensuing network loadings from the second half of the HCP task data were submitted to classification of the psychological tasks based on the implication of brain networks (l2-penalized support vector machines, multi-class, one-versus-rest). l2-contrained support vector machines were employed to choose the most discriminatory network variables by a classical univariate test in a discrete fashion rather than by soft variable selection based on l1 penalization (cf. methods section). This diagnostic analysis (right) revealed the most distinctive k = 1, 5, 10, and 20 network features (red cubes) for each experimental condition of the task battery (cf. Fig 3). The thus discretely selected network features per task were then fed into supervised multi-task classification as a feature space of activity-map-wise continuous activity values. The color intensity of the k cubes quantifies how often the corresponding brain network was selected as important for a task across cross-validation folds. This diagnostic test performed inference on a) the single most discriminative network for each task at k = 1, b) the network variables that get added step-by-step to the feature space of network implications with increasing k, and c) what network variables are unspecific (i.e., not selected) for a given task at k = 20. See Table 1 of the manuscript body for descriptions of task 1 to 18. (PNG) [file pcbi.1004994.s001.png]

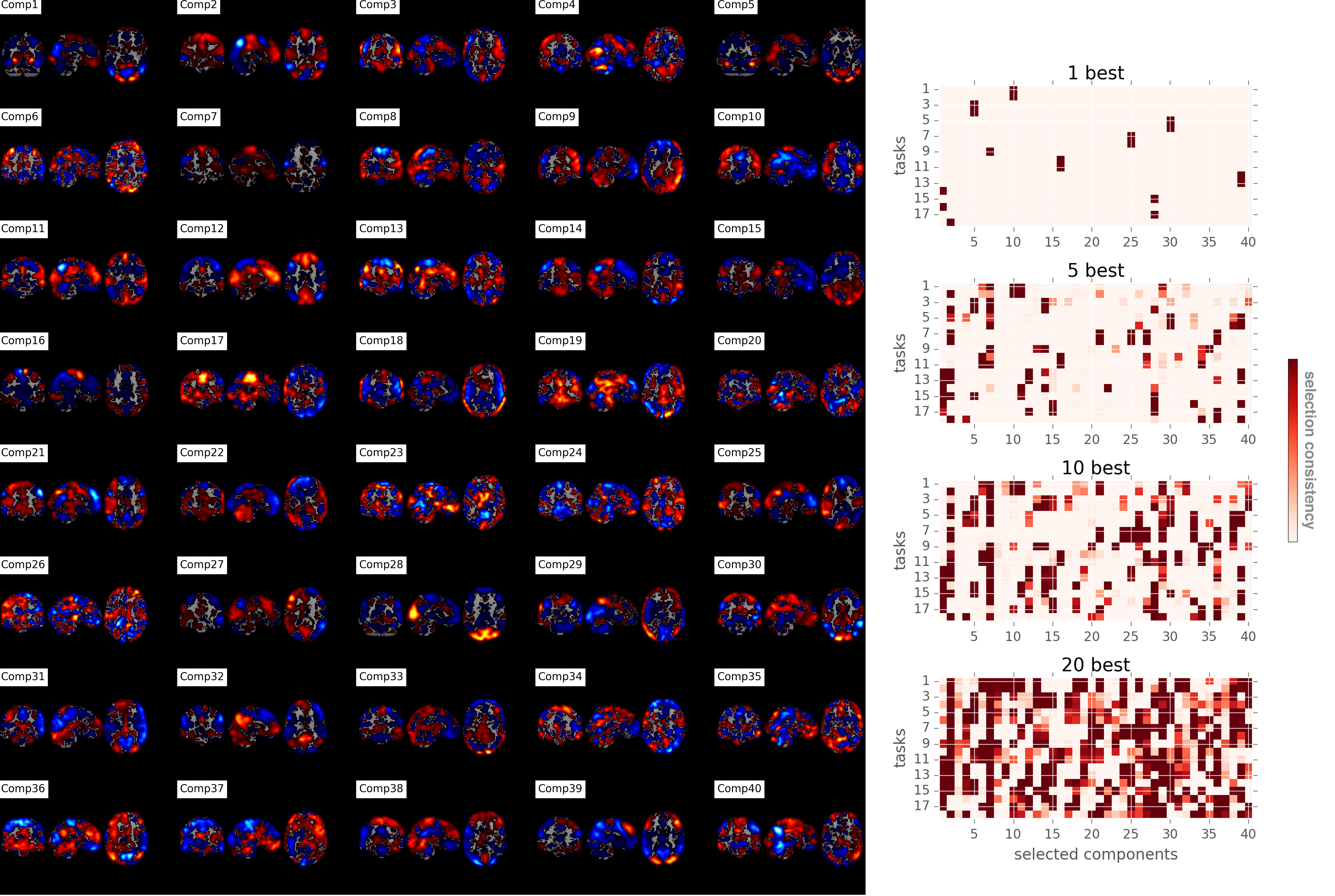

Supplement: S2 Fig — 40 network components underlying 18 HCP tasks (left) have been discovered by ICA (Comp1-40). The ensuing network loadings from the second half of the HCP task data were submitted to classification of the psychological tasks based on the implication of brain networks (l2-penalized support vector machines, multi-class, one-versus-rest). l2-contrained support vector machines were employed to choose the most discriminatory network variables by a classical univariate test in a discrete fashion rather than by soft variable selection based on l1 penalization (cf. methods section). This diagnostic analysis (right) revealed the most distinctive k = 1, 5, 10, and 20 network features (red cubes) for each experimental condition of the task battery (cf. Fig 3). The thus discretely selected network features per task were then fed into supervised multi-task classification as a feature space of activity-map-wise continuous activity values. The color intensity of the k cubes quantifies how often the corresponding brain network was selected as important for a task across cross-validation folds. This diagnostic test performed inference on a) the single most discriminative network for each task at k = 1, b) the network variables that get added step-by-step to the feature space of network implications with increasing k, and c) what network variables are unspecific (i.e., not selected) for a given task at k = 20. See Table 1 of the manuscript body for descriptions of task 1 to 18. (PNG) [file pcbi.1004994.s002.png]

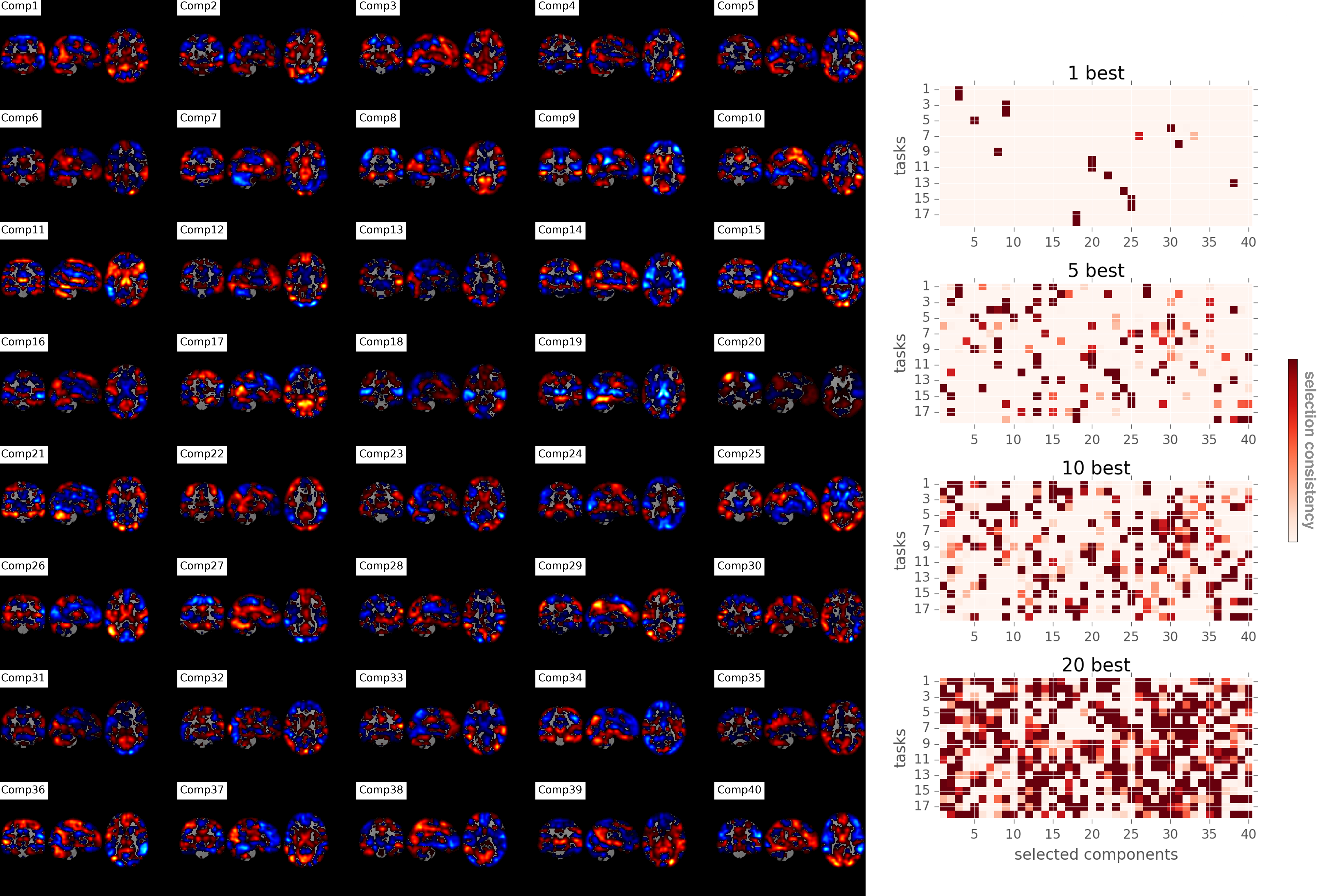

Supplement: S3 Fig — 40 network components underlying 18 ARCHI tasks (left) have been discovered by ICA (Comp1-40). The ensuing network loadings from the second half of the ARCHI task data were submitted to classification of the psychological tasks based on the implication of brain networks (l2-penalized support vector machines, multi-class, one-versus-rest). l2-contrained support vector machines were employed to choose the most discriminatory network variables by a classical univariate test in a discrete fashion rather than by soft variable selection based on l1 penalization (cf. methods section). This diagnostic analysis (right) revealed the most distinctive k = 1, 5, 10, and 20 network features (red cubes) for each experimental condition of the task battery (cf. Fig 3). The thus discretely selected network features per task were then fed into supervised multi-task classification as a feature space of activity-map-wise continuous activity values. The color intensity of the k cubes quantifies how often the corresponding brain network was selected as important for a task across cross-validation folds. This diagnostic test performed inference on a) the single most discriminative network for each task at k = 1, b) the network variables that get added step-by-step to the feature space of network implications with increasing k, and c) what network variables are unspecific (i.e., not selected) for a given task at k = 20. See Table 2 of the manuscript body for descriptions of task 1 to 18. (PNG) [file pcbi.1004994.s003.png]

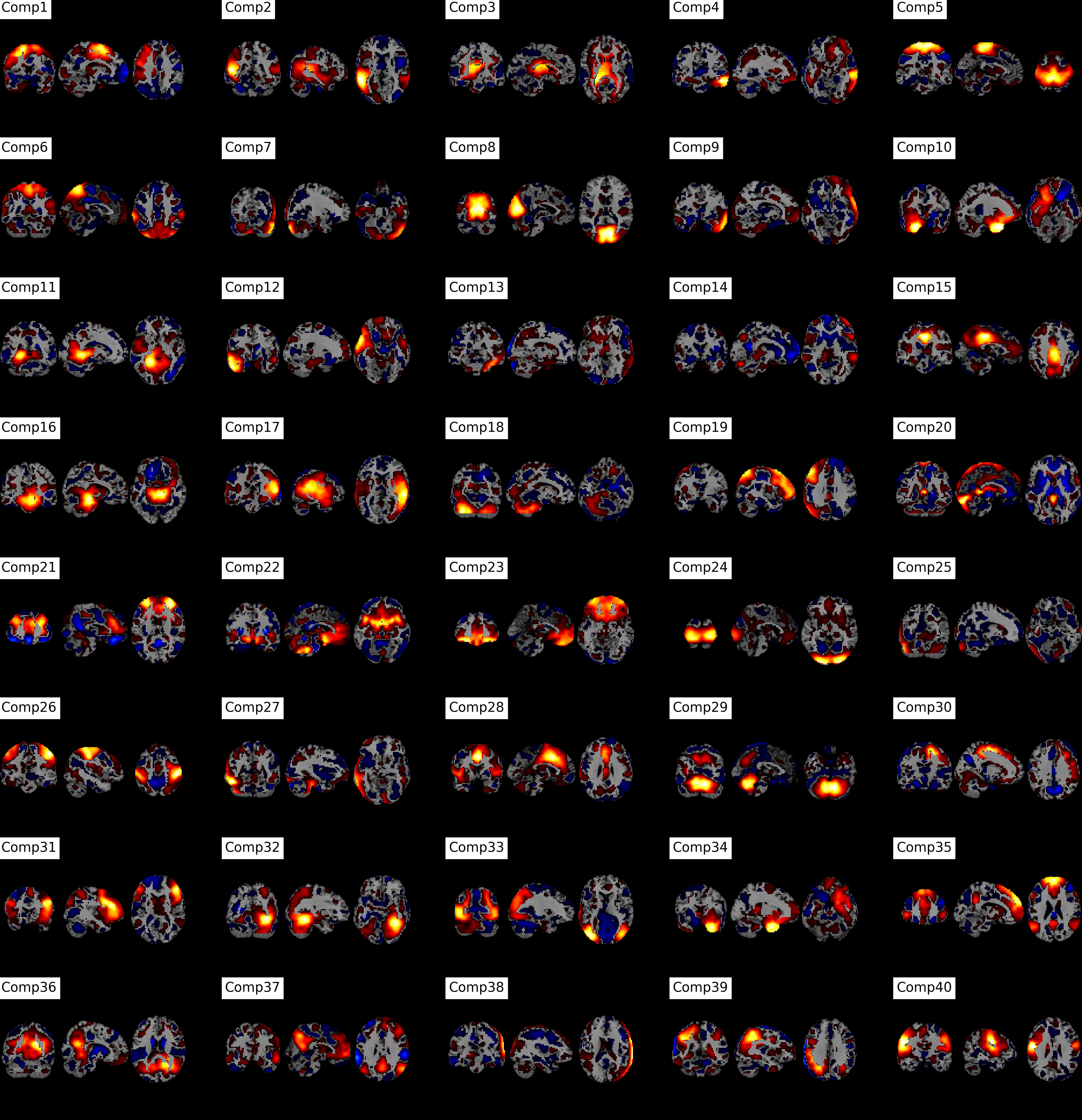

Supplement: S4 Fig — Sparse PCA decomposition was used to derive the 40 most important modes of variation (Comp1-40) in the rest data (cf. methods section). These parsimonious spatial patterns are depicted in coronal, sagittal, and axial slices rendered on the Colin MNI template. Combinations of this dictionary of overlapping major brain networks were used to explain task-evoked neural activity patterns. (PNG) [file pcbi.1004994.s004.png]

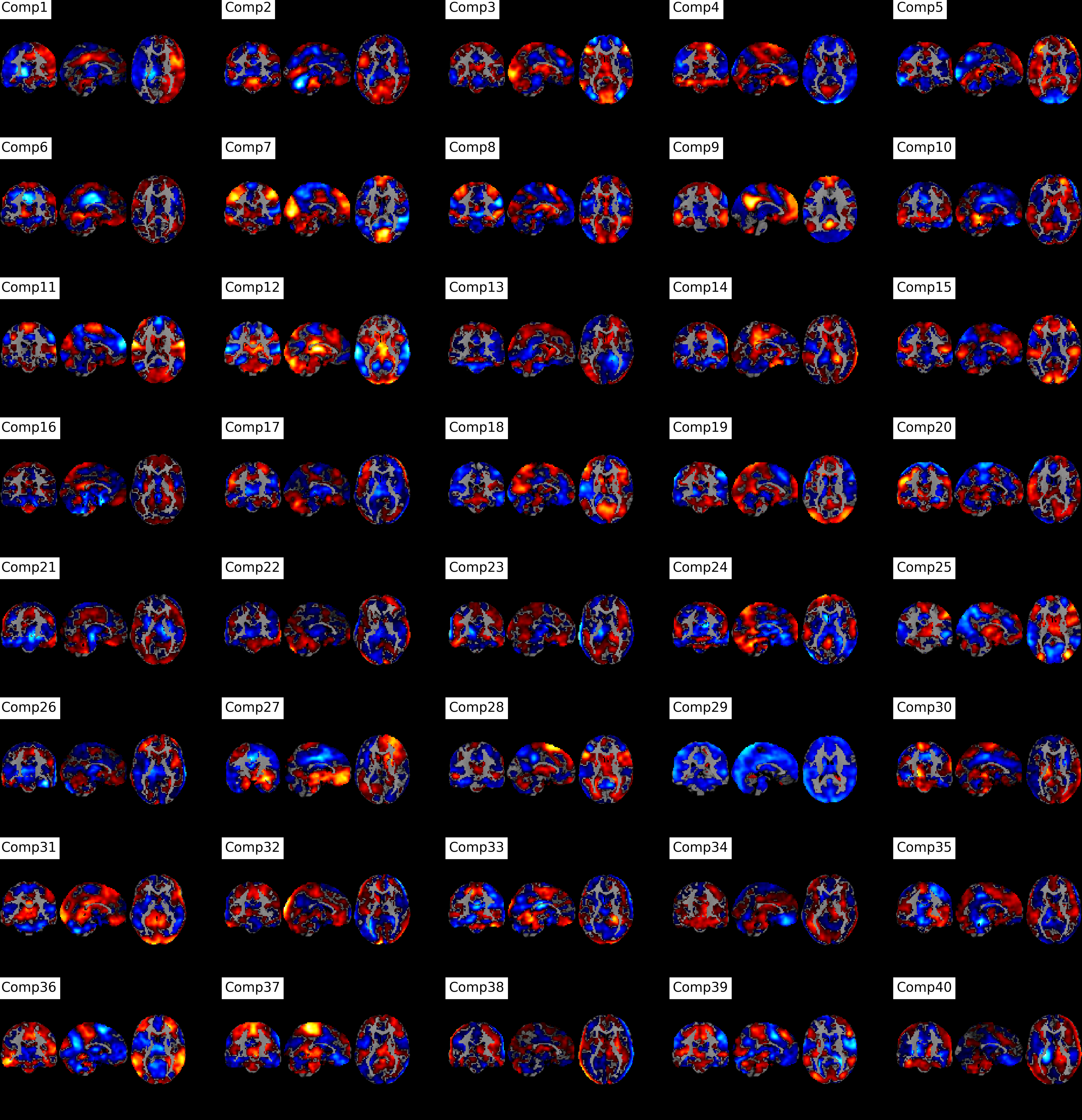

Supplement: S5 Fig — ICA decomposition was used to derive the 40 most important modes of variation (Comp1-40) in the rest data (cf. methods section). These independent spatial patterns are depicted in coronal, sagittal, and axial slices rendered on the Colin MNI template. Combinations of this dictionary of overlapping major brain networks were used to explain task-evoked neural activity patterns. (PNG) [file pcbi.1004994.s005.png]

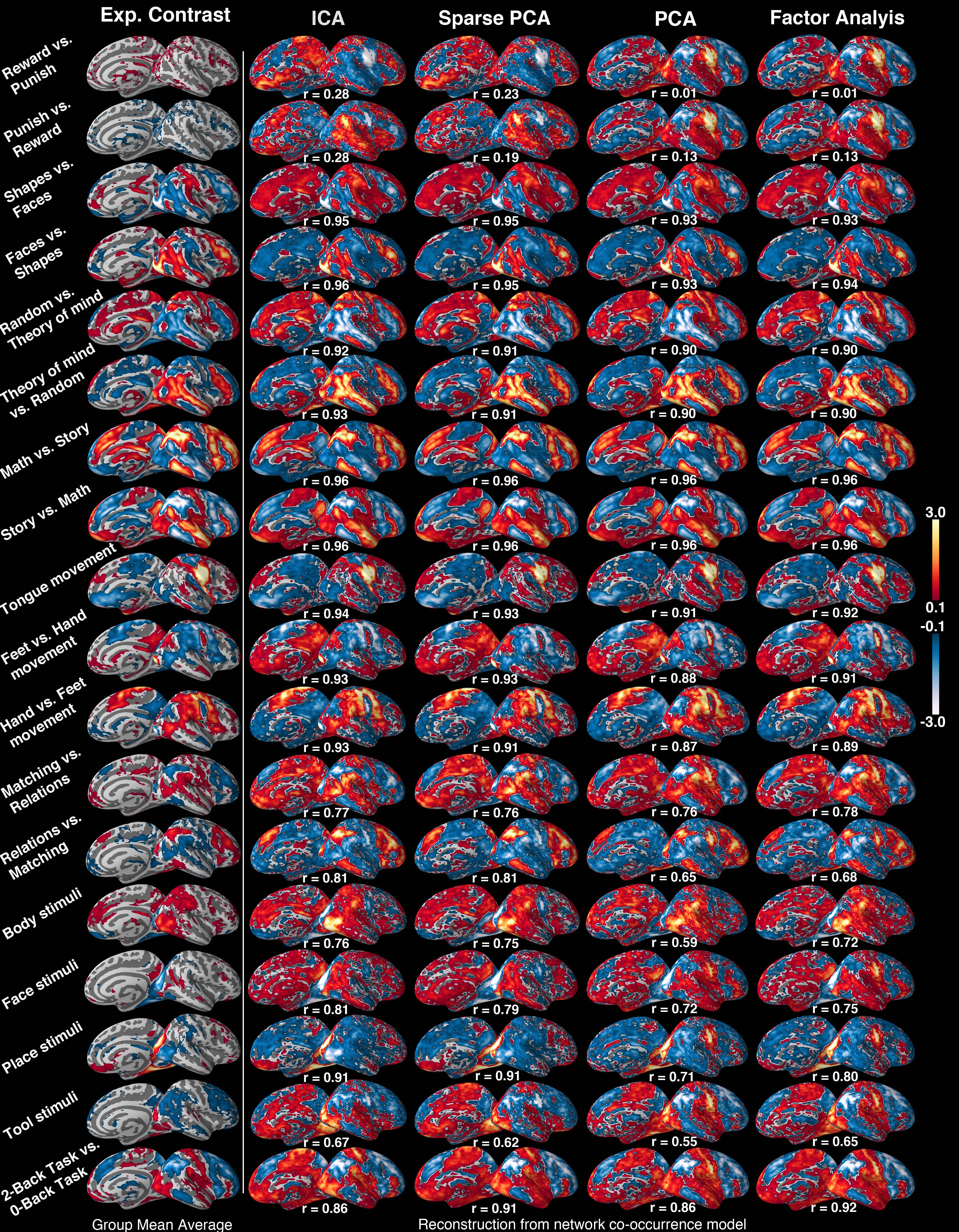

Supplement: S6 Fig — Leftmost column: Average whole-brain activity from 18 experimental tasks of the HCP dataset. The voxel-wise mean was computed across the first-level contrast activity maps from 498 participants. Right columns: Four different network models were derived from the task activity during the 18 HCP tasks. First, four different network decompositions (i.e., ICA, sparse PCA, PCA, and factor analysis) were applied to the first half of the HCP task data. Second, the ensuing sets of 40 brain networks served as a basis for feature engineering to automatically learn distinguishing the 18 tasks in the second data half based on network loadings alone. Third, the quantitative models of task-specific network loadings allowed generating a synthetic whole-brain activity map for each experimental task. The correlation values r quantify the voxel-wise similarity between the reconstructed activity map and the average activity map for each task and network decomposition method. This measure of recovery performance indicates the information loss incurred when first expressing activity maps as 40 network loading values and then translating these values back into whole-brain space. The similarity between real HCP task maps (leftmost column) and synthetic model-derived task maps (right columns) indicates that 40 network loadings can well describe task-evoked neural activity patterns in the HCP task battery. (JPG) [file pcbi.1004994.s006.jpg]

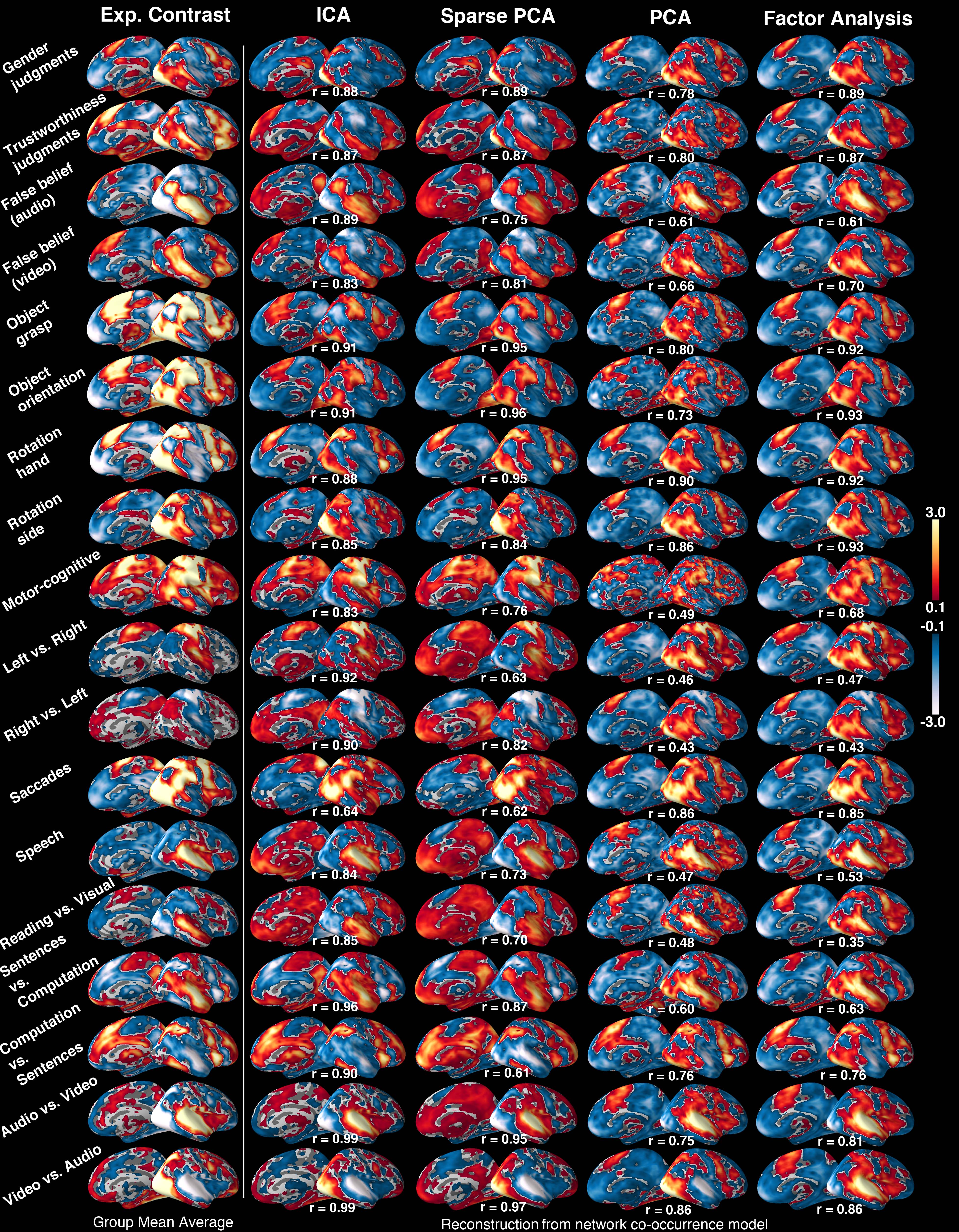

Supplement: S7 Fig — Leftmost column: Average whole-brain activity from 18 experimental tasks of the ARCHI dataset. The voxel-wise mean was computed across the first-level contrast activity maps from 78 participants. Right columns: Four different network models were derived from the task activity during the 18 ARCHI tasks. First, four different network decompositions (i.e., ICA, sparse PCA, PCA, and factor analysis) were applied to the first half of the HCP task data. Second, the ensuing sets of 40 brain networks served as a basis for feature engineering to automatically learn distinguishing the 18 tasks in the second data half based on network loadings alone. Third, the quantitative models of task-specific network loadings allowed generating a synthetic whole-brain activity map for each experimental task. The correlation values r quantify the voxel-wise similarity between the reconstructed activity map and the average activity map for each task and network decomposition method. This measure of recovery performance indicates the information loss incurred when first expressing activity maps as 40 network loading values and then translating these values back into whole-brain space. The similarity between real HCP task maps (leftmost column) and synthetic model-derived task maps (right columns) indicates that 40 network loadings can well describe task-evoked neural activity patterns in the ARCHI battery. (JPG) [file pcbi.1004994.s007.jpg]

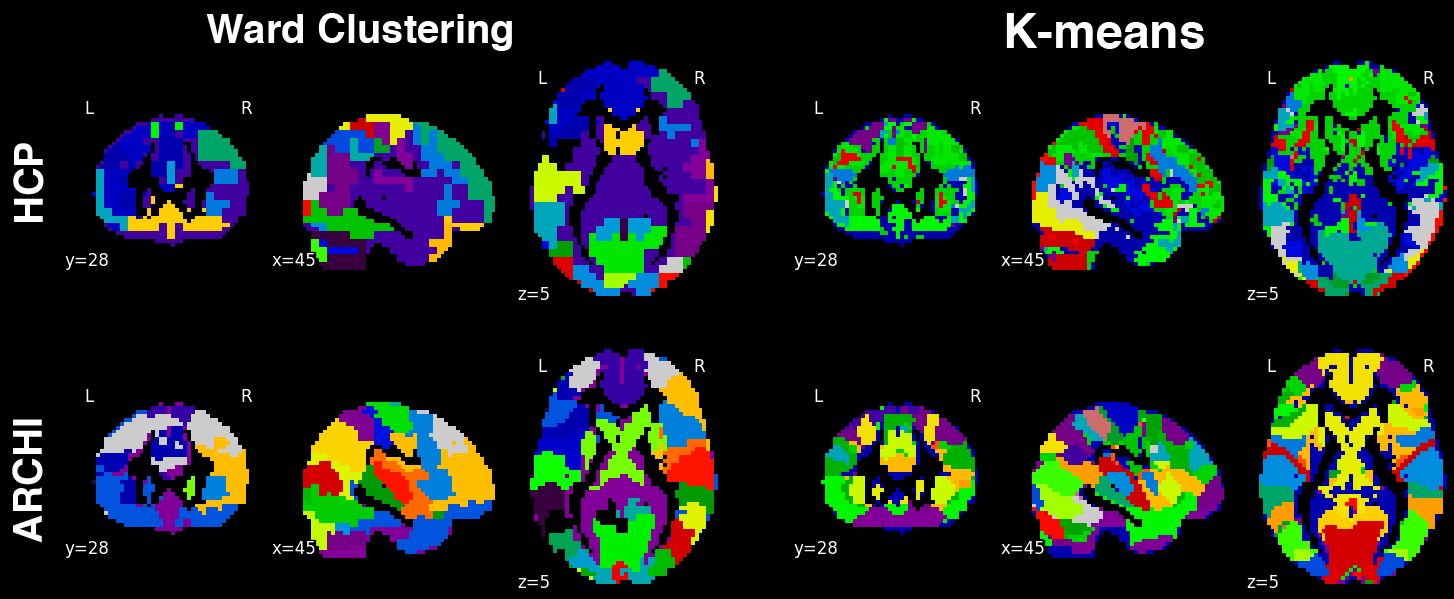

Supplement: S8 Fig — 40 clusters of homogeneous neural activity patterns across tasks and across participants. Spatially constrained ward and spatially unconstrained k-means clustering were applied to the first half of the task maps from the HCP and ARCHI dataset. Each cluster is depicted in a unique, arbitrary color. This grey-matter atlas of non-overlapping brain regions was used to explain task-evoked neural activity patterns. (PNG) [file pcbi.1004994.s008.png]

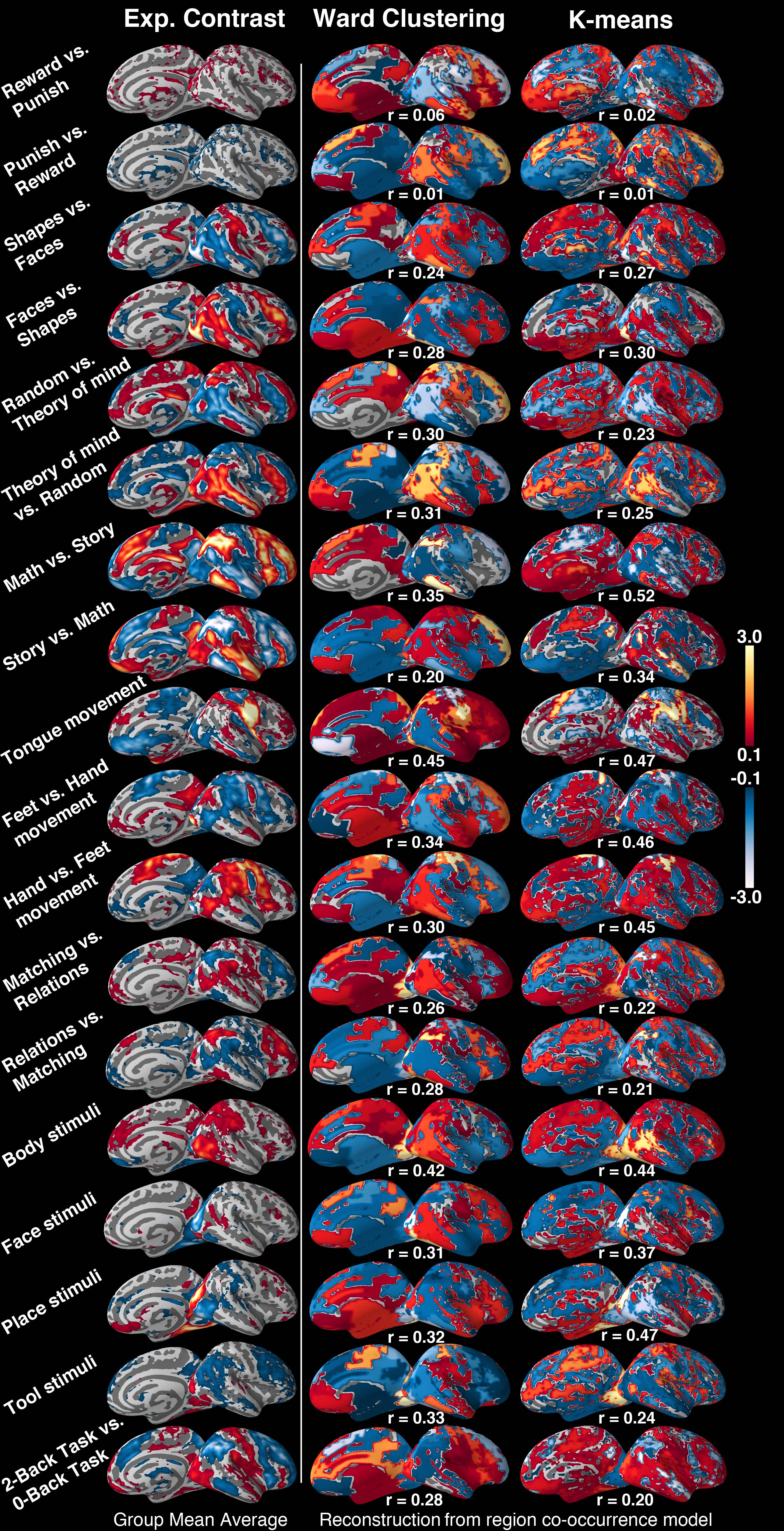

Supplement: S9 Fig — Leftmost column: Average whole-brain activity from 18 experimental tasks of the HCP dataset. The voxel-wise mean was computed across the first-level contrast activity maps from 498 participants. Right columns: Two different region models were derived to explain the task activity during the 18 HCP tasks. First, two different clustering algorithms (i.e., ward and k-means clustering) were applied to the entirety of the HCP task data. Second, the ensuing sets of 40 coherent activation clusters served as a basis for feature engineering to automatically learn to distinguish the 18 tasks based on cluster loadings alone. Third, the quantitative models of task-specific cluster loadings allowed generating a synthetic whole-brain activity map for each experimental task. The correlation values r quantify the voxel-wise similarity between the reconstructed activity map and the average activity map for each task and region clustering method. This measure of recovery performance indicates the information loss incurred when first expressing activity maps as 40 region summary values and then translating these values back into whole-brain space. The frequently low similarity between real HCP task maps (leftmost column) and synthetic model-derived task maps (right columns) indicates that 40 cluster loadings might not well describe task-evoked neural activity patterns in the HCP battery. (JPG) [file pcbi.1004994.s009.jpg]

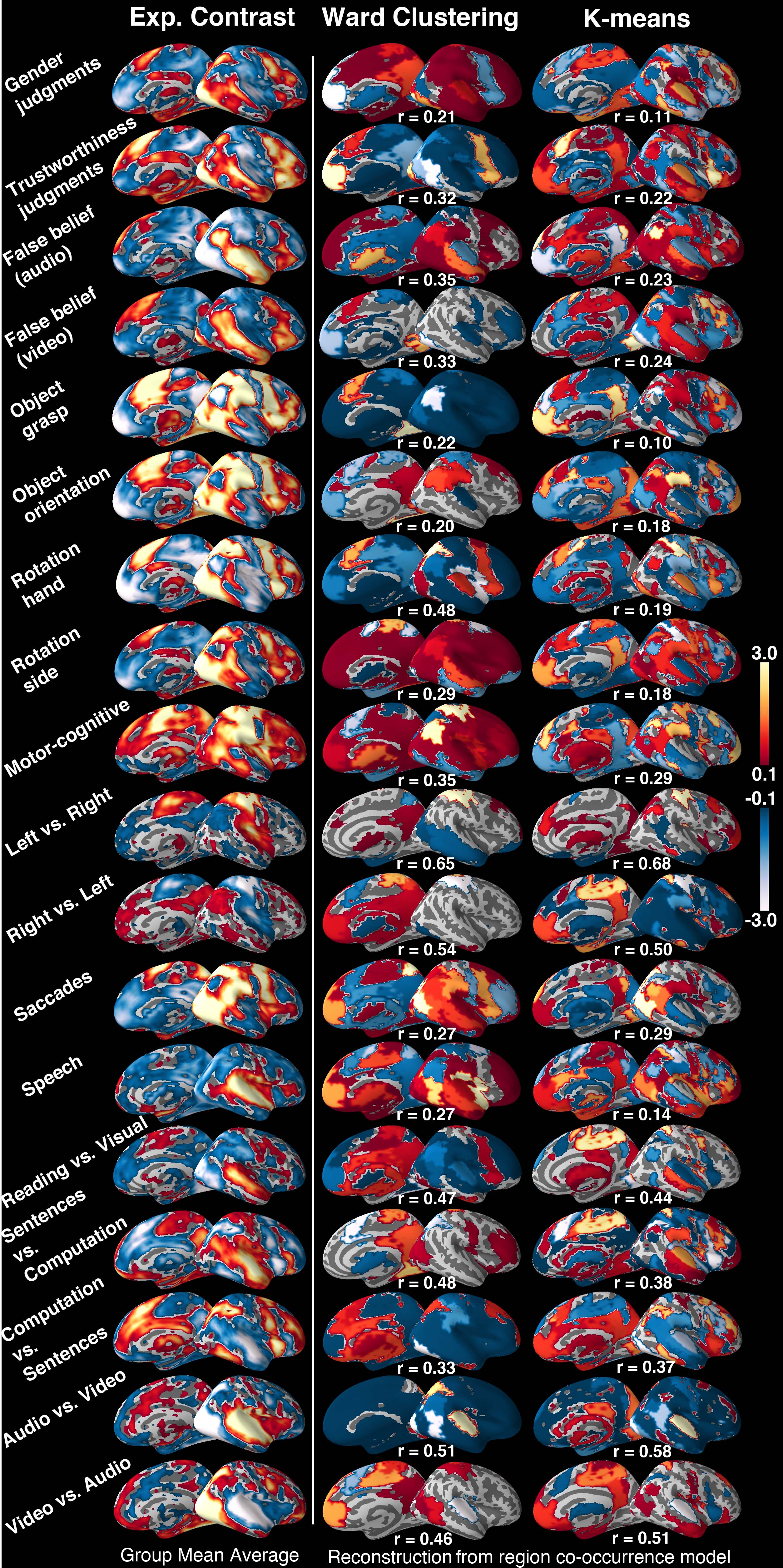

Supplement: S10 Fig — Leftmost column: Average whole-brain activity from 18 experimental tasks of the ARCHI dataset. The voxel-wise mean was computed across the first-level contrast activity maps from 78 participants. Right columns: Two different region models were derived to explain the task activity during the 18 ARCHI tasks. First, two different clustering algorithms (i.e., ward and k-means clustering) were applied to the entirety of the ARCHI task data. Second, the ensuing sets of 40 coherent activation clusters served as a basis for feature engineering to automatically learn distinguishing the 18 tasks based on cluster loadings alone. Third, the quantitative models of task-specific cluster loadings allowed generating a synthetic whole-brain activity map for each experimental task. The correlation values r quantify the voxel-wise similarity between the reconstructed activity map and the average activity map for each task and region clustering method. This measure of recovery performance indicates the information loss incurred when first expressing activity maps as 40 region summary values and then translating these values back into whole-brain space. The frequently low similarity between real ARCHI task maps (leftmost column) and synthetic model-derived task maps (right columns) indicates that 40 cluster loadings might not well describe task-evoked neural activity patterns in the ARCHI battery. (JPG) [file pcbi.1004994.s010.jpg]

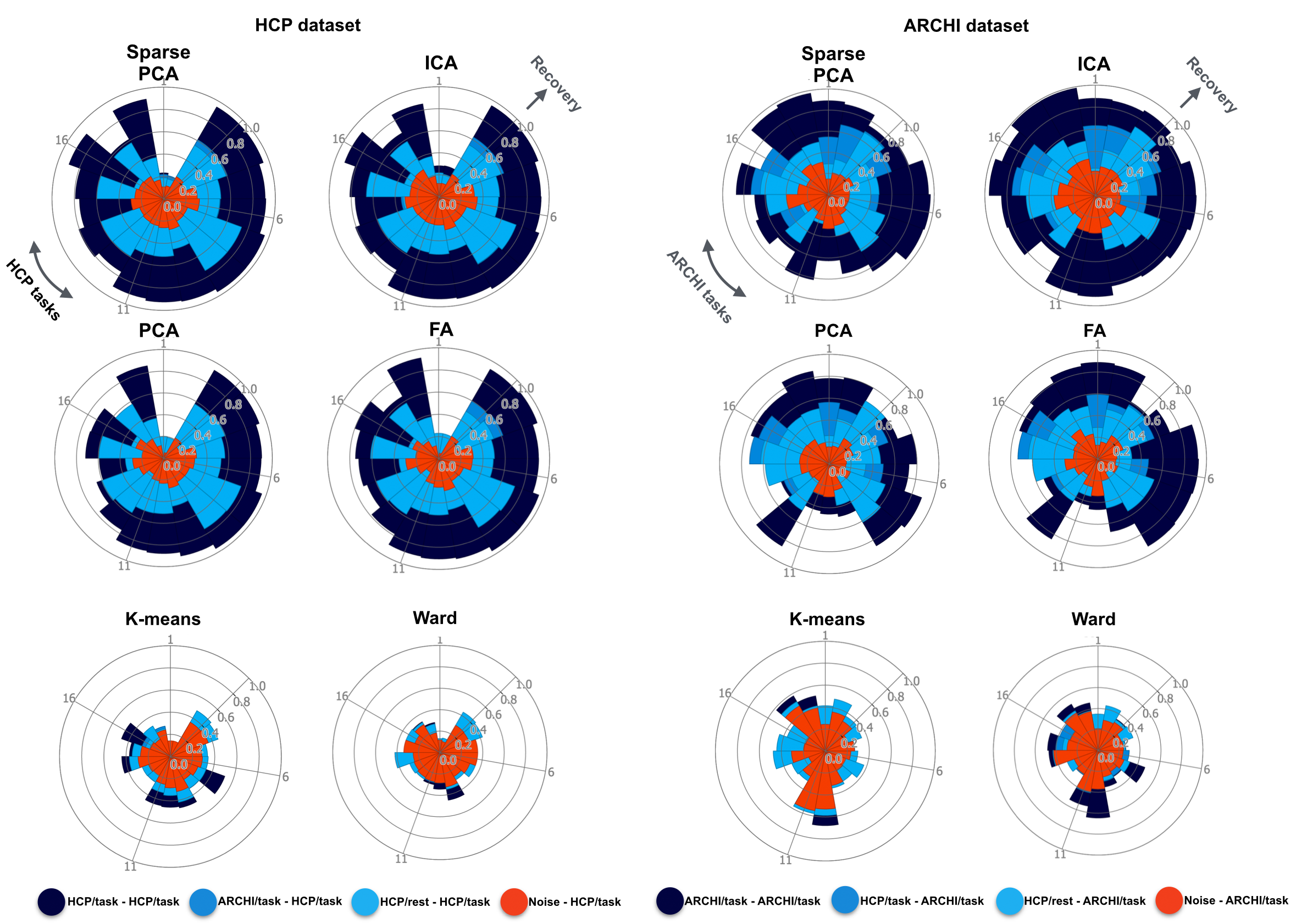

Supplement: S11 Fig — Four different network co-occurrence models (upper and middle row) were computed based on decomposition based on sparse PCA, ICA, PCA, and factor analysis (FA). They capture functional brain architecture with emphasis on functional integration as opposed to regional specialization. Two different region co-occurrence models (lower row) were computed based on ward clustering (regions always spatially connected) and k-means clustering (no spatial constraint). They capture functional brain architecture with emphasis on regional specialization. The recovery performance of all 18 tasks (radial columns) is measured by the Pearson correlation r between the model-derived task activity maps and the average first-level task map. As a first conclusion, modeling task-specific neural activity appears to be more successful based on functional network units than on functional region units. Additionally, network and region dictionaries were derived from i) identical task-data as positive test (dark blue), ii) non-identical task-data (medium blue), iii) resting-state data (light blue), and iv) Gaussian noise as negative test (red). As a second conclusion, network dictionaries derived from non-identical task maps and rest maps are similarly successful in recovering whole-brain activity during divering experimental tasks. This was confirmed by univariate t-tests between the task-wise correlation values r (cf. results section). (PNG) [file pcbi.1004994.s011.png]

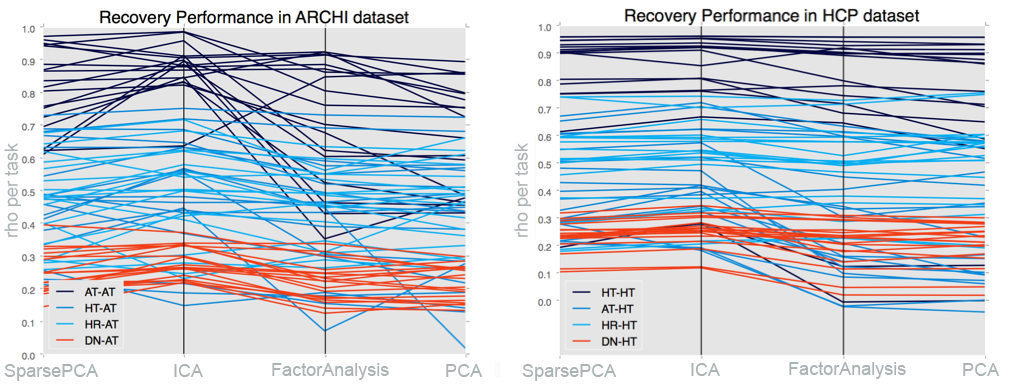

Supplement: S12 Fig — Parallel coordinate plots that depict the recovery performance by one line for each of the 18 tasks in both datasets. The network dictionaries were derived from i) identical task-data (dark blue, AT = ARCHI task data, HT = HCP task data), ii) non-identical task-data (medium blue), iii) resting-state data (light blue, HR = HCP rest data), and iv) Gaussian noise (red, DN = data noise), analogous to S11 Fig. An important conclusion is that network dictionaries derived from non-identical task maps and rest maps are similarly successful in recovering whole-brain activity during divering experimental tasks. This was confirmed by univariate t-tests between the task-wise correlation values r (cf. results section). (PNG) [file pcbi.1004994.s012.png]
